# Increased intrinsic membrane excitability is associated with olivary hypertrophy in spinocerebellar ataxia type 1

**DOI:** 10.1093/hmg/ddae146

**Published:** 2024-10-30

**Authors:** Logan M Morrison, Haoran Huang, Hillary P Handler, Min Fu, Deborah M Jones, David D Bushart, Samuel S Pappas, Harry T Orr, Vikram G Shakkottai

**Affiliations:** Neuroscience Graduate Program, University of Michigan, Ann Arbor, MI 48109, United States; Peter O’Donnell Jr. Brain Institute, University of Texas Southwestern Medical Center, 6124 Harry Hines Blvd. Dallas, TX 75390, United States; Department of Neurology, University of Texas Southwestern Medical Center, Dallas, 5323 Harry Hines Blvd., TX 75390, United States; Medical Scientist Training Program, The Ohio State University, Columbus, OH 43210, United States; College of Medicine, The Ohio State University, Columbus, OH 43210, United States; Molecular Diagnostics Laboratory, University of Minnesota Fairview Medical Center, Minneapolis, MN 55455, United States; Peter O’Donnell Jr. Brain Institute, University of Texas Southwestern Medical Center, 6124 Harry Hines Blvd. Dallas, TX 75390, United States; Department of Neurology, University of Texas Southwestern Medical Center, Dallas, 5323 Harry Hines Blvd., TX 75390, United States; Department of Neurology, University of Texas Southwestern Medical Center, Dallas, 5323 Harry Hines Blvd., TX 75390, United States; College of Medicine, The Ohio State University, Columbus, OH 43210, United States; Peter O’Donnell Jr. Brain Institute, University of Texas Southwestern Medical Center, 6124 Harry Hines Blvd. Dallas, TX 75390, United States; Department of Neurology, University of Texas Southwestern Medical Center, Dallas, 5323 Harry Hines Blvd., TX 75390, United States; Institute for Translational Neuroscience, University of Minnesota, Minneapolis, 420 Delaware Street SE, MN 55455, United States; Department of Laboratory Medicine and Pathology, University of Minnesota, 420 Delaware Street SE, Minneapolis, MN 55455, United States; Peter O’Donnell Jr. Brain Institute, University of Texas Southwestern Medical Center, 6124 Harry Hines Blvd. Dallas, TX 75390, United States; Department of Neurology, University of Texas Southwestern Medical Center, Dallas, 5323 Harry Hines Blvd., TX 75390, United States

**Keywords:** ion channel, inferior olivary nucleus, cerebellar ataxia, SCA

## Abstract

One of the characteristic regions of brainstem degeneration across multiple spinocerebellar ataxias (SCAs) is the inferior olive (IO), a medullary nucleus that plays a key role in motor learning. The vulnerability of IO neurons remains a poorly-understood area of SCA pathology. In this work, we address this by evaluating IO disease in SCA1, a prototypic inherited olivopontocerebellar atrophy, using the genetically-precise SCA1 knock-in (SCA1-KI) mouse. We find that these mice exhibit olivary hypertrophy, a phenotype reminiscent of a degenerative disorder known as hypertrophic olivary degeneration (HOD). Similar to early stages of HOD, SCA1-KI IO neurons display early dendritic lengthening and later somatic expansion without frank cell loss. Though HOD is known to be caused by brainstem lesions that disrupt IO inhibitory innervation, we observe no loss of inhibitory terminals in the SCA1-KI IO. Additionally, we find that a separate mouse model of SCA1 in which mutant ATXN1 is expressed solely in cerebellar Purkinje cells shows no evidence of olivary hypertrophy. Patch-clamp recordings from brainstem slices indicate that SCA1-KI IO neurons are hyperexcitable, generating spike trains in response to membrane depolarization. Transcriptome analysis further reveals reduced medullary expression of ion channels responsible for IO neuron spike afterhyperpolarization (AHP)—a result that appears to have a functional consequence, as SCA1-KI IO neuron spikes exhibit a diminished AHP. These findings suggest that expression of mutant ATXN1 in IO neurons results in an HOD-like olivary hypertrophy, in association with increased intrinsic membrane excitability and ion channel transcriptional dysregulation.

## Graphical Abstract

**Graphical Abstract:**
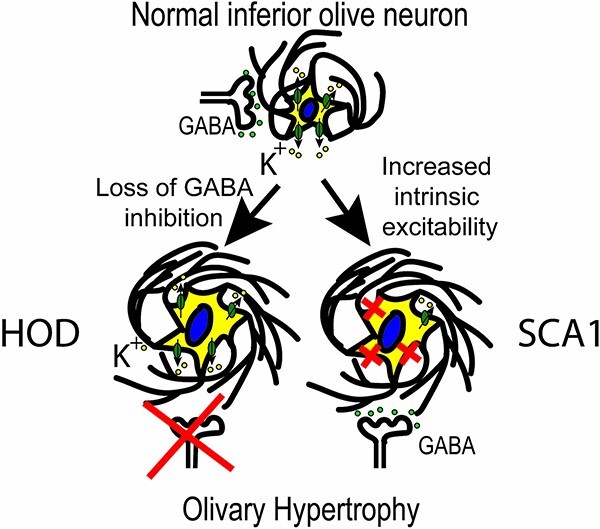

Graphical abstract showing that, canonically, olivary neuron hypertrophy is due to loss of inhibitory GABA input to the inferior olivary nucleus. We show for the first time that olivary neuron hypertrophy occurs in SCA1 in association with increased intrinsic excitability and loss of potassium channel function, and in the absence of overt inhibitory deafferentation of the inferior olivary nucleus.

## Introduction

The inferior olive (IO) is a nucleus in the medullary brainstem that plays an important role in cerebellar motor learning [1]. IO neurons send powerful excitatory projections known as “climbing fibers” into the cerebellum, where they synapse onto Purkinje cell dendrites to generate cerebellar complex spikes [2–5]. The IO is susceptible to degeneration in a number of conditions, including sporadic olivopontocerebellar atrophy (OPCA) [6, 7] and the spinocerebellar ataxias [8]. IO neurons are also vulnerable to a pathology known as hypertrophic olivary degeneration (HOD). Clinically, HOD occurs secondary to lesions in the brainstem (pontine hemorrhage, most commonly [9]) that disrupt inhibitory fibers that travel from the contralateral dentate nucleus of the cerebellum and through the brainstem to synapse onto the IO (i.e., via the central tegmental tract within the Guillain-Mollaret triangle [10]). An acute lesion to these fibers results in an initial increase in IO neuron soma size, generally observed as an increase in gross IO volume [11–13]. This is ultimately followed by cell loss, gliosis, and atrophy of the IO, a process that sometimes extends for years after the initial insult [14, 15]. HOD is not unique to humans: hemicerebellectomy in cats results in olivary hypertrophy with the characteristic features of increased soma size of neurons of the contralateral inferior olivary nucleus, causing macroscopic enlargement of the IO [12, 16–18]. Although HOD ultimately results in IO degeneration, its pathology is considered distinct from the IO atrophy observed in OPCA and the spinocerebellar ataxias due to the lack of any clinically-evident IO hypertrophy in these disorders [19].

The spinocerebellar ataxias (SCAs) are a group of inherited neurodegenerative diseases that cause a progressive loss of motor function [8, 20]. Progressive brainstem degeneration (including in the IO) occurs in SCA1, SCA2, SCA3, and SCA7, which together account for the majority of SCA cases [21]. This pathology manifests late in disease progression, corresponding closely to the onset of breathing and swallowing deficits (symptoms that can eventually cause premature death, especially in SCA1) [20]. Among these common SCAs, the fastest-progressing is SCA1 [22], a subtype that exhibits IO degeneration as a characteristic pathology [23]. The mechanisms that underlie this degeneration, as well as brainstem pathology in the SCAs broadly, remain poorly understood.

In this study, we examined IO morphology and physiology in the genetically-precise SCA1 knock-in (SCA1-KI) mouse model, which expresses an expanded CAG triplet repeat in the endogenous *Atxn1* locus [24]. For the first time, we have identified olivary hypertrophy reminiscent of early HOD in a model not of brainstem injury, but of neurodegenerative disease. In neurons of the principal olivary nucleus (IOPr) of SCA1-KI mice, we observe early dendritic hypertrophy followed by late somatic hypertrophy. We find that these morphological changes are concurrent with a loss of immunoreactivity for the calcium-binding protein calbindin. Calbindin loss is characteristic of SCA1 pathology in both IO neurons [23, 25] and Purkinje cells [26, 27] and has been used as a surrogate for neurodegeneration in various mouse models of the disease, including SCA1-KI mice [24, 28]. Interestingly, structural and functional analyses of IO innervation demonstrate no gross loss of inhibitory input, suggesting an intrinsic mechanism of HOD in SCA1 that is distinct from the extrinsic mechanism produced by lesions of the brainstem. Furthermore, using patch-clamp electrophysiology, we find that SCA1-KI IO neurons are also hyperexcitable. Unbiased transcriptome analysis demonstrates that this hyperexcitability is likely due to the reduced expression of genes encoding the calcium and potassium channels that mediate spike afterhyperpolarization (AHP), thereby regulating IO neuron intrinsic excitability [29, 30]. Together, these results suggest that increased membrane excitability is a potential link between disparate causes of olivary hypertrophy and, potentially, the degenerative hypertrophy seen in HOD.

## Materials and methods

### Mouse studies

All animal studies were reviewed and approved by the Institutional Animal Care and Use Committee (IACUC) at the institution where they were performed (University of Michigan, University of Texas Southwestern Medical Center, or University of Minnesota) and were conducted in accordance with the United States Public Health Service’s Policy on Humane Care and Use of Laboratory Animals. SCA1 knock-in (SCA1-KI) mice (RRID:MGI:3774931), which express an expanded CAG triplet repeat in the endogenous *Atxn1* locus [24], were maintained on a C57BL/6 background. SCA1-KI mice were heterozygous for the expanded *Atxn1* allele (*Atxn1^154Q/2Q^*), with wild-type littermates (*Atxn1^2Q/2Q^*) used as controls. SCA1 transgenic (SCA1-Tg) mice (RRID:MGI:5518618) overexpress the human *ATXN1* gene with an expanded CAG triplet repeat under the Purkinje cell-specific murine *Pcp2 (L7)* promoter [28] and were maintained on an FVB background (Jackson Labs, RRID:IMSR_JAX:001800). SCA1-Tg mice were homozygous for the transgene (*ATXN1[82Q]^tg/tg^*), with age/sex-matched wild-type FVB mice used as controls. For both mouse models, studies were performed at either 13–15 weeks of age (defined as the “14 week” timepoint) or 29–32 weeks of age (defined as the “30 week” timepoint). Sexes were balanced for all animal studies.

### Patch-clamp electrophysiology

#### Solutions

Artificial cerebrospinal fluid (aCSF) used in these experiments contained: 125 mM NaCl, 3.8 mM KCl, 26 mM NaHCO_3_, 1.25 mM NaH_2_PO_4_, 2 mM CaCl_2_, 1 mM MgCl_2_, and 10 mM glucose. For all recordings, pipettes were filled with internal solution containing: 140 mM K-Gluconate, 4 mM NaCl, 10^−3^ mM CaCl_2_, 4 mM Mg-ATP, 10^−2^ mM EGTA, 10 mM HEPES, at pH 7.3 and osmolarity ~285 mOsm, as described previously for IO recordings [31].

#### Preparation of brain slices for acute electrophysiological recordings

Slices were prepared using the “hot cut” technique [32]. This process, which involves tissue sectioning in standard aCSF at physiological temperatures rather than a sucrose-rich aCSF at near-freezing temperatures, is a recent advancement that has allowed for patch-clamp recordings in brain regions previously deemed inaccessible in adult mice due to their hypersensitivity to slicing-induced damage. This technique has facilitated studies of nuclei never-before investigated in the adult mouse brain, including the inferior olive [31]. Briefly, mice were deeply anesthetized by isoflurane inhalation, decapitated, and brains were rapidly removed and submerged in pre-warmed (33°C) aCSF. 300 μm coronal slices were prepared on a VT1200 vibratome (Leica Biosystems, Deer Park, IL) in aCSF held at 32.5°C–34.0°C during sectioning. Slices were then incubated in carbogen-bubbled (95% O2, 5% CO2) aCSF at 33°C for 45 min. Slices were then stored in carbogen-bubbled aCSF at room temperature (RT) until use. During recording, slices were placed in a recording chamber and continuously perfused with carbogen-bubbled aCSF at 33°C at a flow rate of 2.5 ml/min.

#### Patch-clamp recordings

IO neurons were visually identified for patch-clamp recordings using a 40× water immersion objective and a Nikon Eclipse FN1 upright microscope with infrared differential interference contrast (IR-DIC) optics. Identified cells were visualized using NIS Elements image analysis software (Nikon, Melville, NY). Patch pipettes were pulled to resistances of ~3 MΩ from thin-walled glass capillaries (World Precision Instruments, Cat# 1B120F-4). Importantly, we found that pipette shape had an outsized impact on the success of these difficult recordings; as such, special care was taken to make pipettes with a short taper and a perfectly flat, ~1 μm diameter tip. Data were acquired using a CV-7B headstage amplifier, a Multiclamp 700B amplifier, a Digidata 1440A interface (Axon Instruments, San Jose, CA), and pClamp-10 software (Molecular Devices, San Jose, CA). All data were digitized at 100 kHz. All voltages were corrected for liquid gap junction potential, previously calculated to be 10 mV [33]. Current clamp recordings were performed in the bridge-balance mode of a dedicated voltage-following amplifier. Series resistance correction was not routinely employed for voltage-clamp recordings. Series resistance was 11.3 ± 1.2 MΩ for wild-type (n = 16 neurons, 14 mice) and 9.2 ± 4.9 MΩ (n = 16 neurons, 11 mice) for SCA1-KI mice. There was no significant difference in the series resistance between wild-type and SCA1-KI neurons (data displayed as mean ± SEM, Student’s t-test, *P* < 0.05). Specific resistance was calculated as input resistance divided by the cell-capacitance. Cell capacitance was calculated as area under the voltage–current curve from a 10 mV voltage step from −80 mV. Analysis of spike waveforms in current clamp recordings was performed using a custom Matlab script. Spike threshold was calculated as voltage corresponding to 10% of the maximum dV/dt of the rising phase of the action potential. AHP amplitude was calculated from threshold and was determined to be the most negative membrane potential in the post-spike period before the subsequent spike or the end of the current step.

### Purkinje cell *in-vivo* electrophysiology

#### Surgical preparation of craniotomy site

*In-vivo* electrophysiological recordings were performed with custom equipment and techniques as described previously [34]. Briefly: 14-week-old wild-type and SCA1-KI mice were surgically prepared for recordings one day in advance. Isoflurane anesthesia (dosage 4% for induction, 1.5% for maintenance) was used during all surgical procedures. After a scalp incision, two 0.6 × 0.06” SL flat SS anchor screws (Antrin, Fallbrook, CA) were placed into the skull slightly posterior to bregma. A custom titanium headplate was then affixed to both the skull and the anchor screws with C&B-Metabond (Parkell, Edgewood, NY), and a recording chamber was shaped around the skull above the cerebellum using dental cement (Lang Dental, Wheeling, IL). Two 3 mm craniotomies (centered at −6.2 AP, ±2.1 ML from Bregma) were performed to expose the anterior lobules of each cerebellar hemisphere. Craniotomy sites were protected with Kwik-Sil (World Precision Instruments, Sarasota, FL) until recording.

#### Recording procedure

On the day of recording, animals were secured to the recording platform using the titanium headplate described above. All recordings were performed in awake mice in a single session per mouse, with the total duration of the session limited to 1 h. After the headplate was attached to the platform, the dental cement recording chamber was filled with sterile PBS and a 2.5–3.5 MΩ tungsten electrode (Thomas Recording, Giessen, Germany) was inserted into one of the craniotomy sites to acquire data. The recording electrode was slowly lowered into the cerebellar cortex until Purkinje cells were identified by their characteristic physiology (20–80 Hz simple spikes and the presence of complex spikes). From this point, the electrode was lowered in 10 μm increments until maximum simple spike amplitude was reached. Though electrode placement varied between animals, final electrode depth was typically 2–3 mm from the surface of the brain. Purkinje cell activity was recorded using a DP-301 differential amplifier (Warner Instruments, Holliston, MA), digitized at 100 kHz using a Digidata 1440A interface (Axon Instruments, San Jose, CA), and analyzed using pClamp-10 software (Molecular Devices, San Jose, CA). A minimum of 5 min of activity was recorded per craniotomy site. Recording position in the cerebellar cortex was verified by assessing tissue damage from the recording electrode via Nissl staining.

#### Data analysis

Complex spike frequency was analyzed in 10 Hz high-pass filtered traces, with the criterion for identification that complex spikes must exhibit a voltage higher than the maximum simple spike peak for the entire trace, as described previously [35]. Displayed traces from *in-vivo* electrophysiological recordings were first filtered to reduce minor 60 Hz electrical interference, then high-pass filtered at 10 Hz.

### Cell filling and morphological analysis

#### Filling protocol and section preparation

Before patch clamp recordings, Alexa Fluor 488 Succinimidyl Ester (Invitrogen, Cat# A20000) powder was dissolved separately in internal solution at a concentration of 1 mg/ml. Pipette tips were filled with a miniscule amount of standard internal solution so fluorescent dye would not be washed over the tissue when attempting to patch. This was accomplished by placing pipettes upside down (i.e. tip up) in a microcentrifuge tube containing ~20 μl internal solution for ~5 min, which filled the pipette tip by capillary action. From here, pipettes were backfilled with the fluorescent internal solution, allowing cells to be filled with dye while recording. All cells were filled for 15–20 min and confirmed as filled after pipette removal by the presence of intense somatic fluorescence at 40X (as shown in Fig. 5A, inset). Only one cell per side was filled on each section to avoid tracing errors from dendrite overlap. In addition, a scalpel was used to make a small notch in the left spinal trigeminal tract of each section to mark its orientation. After filling was complete, free-floating brainstem sections were immediately fixed in 1% PFA for 1 h at room temperature, then washed 3× in PBS. To avoid crushing the sections during mounting (as patch-clamp slices are much thicker than typical histological sections) a square was drawn on the slide using clear nail polish and allowed to dry immediately before attempting to mount the tissue. Sections were mounted in this square using ProLong Gold Antifade Mounting Medium with DAPI (Cell Signaling Technology, Cat# 8961S), with specific attention being paid to the notch in the section to ensure that the surface of the section with the filled cell was mounted against the coverslip, not the slide.

#### Confocal imaging of filled cells

Sections were imaged using a Nikon C2+ microscope with a 63× oil immersion objective. Imaging of each cell was accomplished by taking multiple z-stack images at the microscope’s minimum of 0.207 μm between each image of the stack.

#### 3D reconstruction of cells and morphological analysis

After imaging, 3D reconstructions of cells were generated from z-stack images using the open-source tracing software Vaa3D [36–38]. For each cell, a conservative initial trace was generated automatically by Vaa3D, then manually reviewed and appended to include all visible projections of the neuron. All single-cell morphological calculations were made automatically by Vaa3D, with dendritic tip and bifurcation counts confirmed as accurate by manual review.

### Immunohistochemistry

#### Sample preparation and imaging

Mice were anesthetized under isoflurane inhalation and brains were removed, fixed in 1% paraformaldehyde (PFA) for 1 h, after which they were placed in 30% sucrose in phosphate-buffered saline (PBS) for 48 h at 4°C. Brains were then cryo-embedded in a 1:1 mixture of 30% sucrose (in PBS) and OCT compound (Fisher Scientific, Cat# 23-730-571) at −80°C for at least 24 h. Tissue blocks were sectioned to 20 μm thickness on a CM1850 cryostat (Leica Biosystems, Deer Park, IL). Tissue was permeabilized with 0.4% triton in PBS, then non-specific binding was minimized by blocking with 5% normal goat serum in 0.1% triton in PBS. Primary antibodies (Abs) were incubated in PBS containing 0.1% triton and 2% normal goat serum at 4°C overnight, while secondary Abs were applied for 1 h at room temperature in PBS.

#### Antibodies used

To stain for neuronal nuclear protein (NeuN) for stereology (Figs 2 and 4), Rabbit anti-NeuN primary Ab (1:500, Cell Signaling, Cat# 12943, RRID:AB_2630395) and Goat anti-Rabbit IgG (H + L) Alexa Fluor 488-conjugated secondary Ab (1:200; Invitrogen, Cat# A11034, RRID:AB_2576217) were used. To stain for Calbindin (Calb) for stereology (Figs 2 and 4), Mouse anti-Calb primary Ab (1:1000; Sigma Aldrich, Cat# C9848, RRID:AB_476894) and Goat anti-Mouse IgG (H + L) Alexa Fluor 594-conjugated secondary Ab (1:200; Invitrogen, Cat# A11005, RRID:AB_2534073) were used. To stain for glutamate decarboxylase (GAD) for assessment of IO inhibitory innervation (Figs 3 and 4), Rabbit anti-GAD primary Ab (1:500, Sigma-Aldrich, Cat# G5163, RRID:AB_477019) and Goat anti-Rabbit IgG (H + L) Alexa Fluor 594-conjugated secondary Ab (1:200, Invitrogen, Cat# A11012, RRID:AB_2534079) were used. To stain for Calbindin (Calb) for assessment of IO inhibitory terminals (Figs 3 and 4), Mouse anti-Calb primary Ab (1:1000; Sigma Aldrich, Cat# C9848, RRID:AB_476894) and Goat anti-Mouse IgG (H + L) Alexa Fluor 488-conjugated secondary Ab (1:200; Invitrogen, Cat# A11001, RRID:AB_2534069) were used. To stain for small conductance calcium-activated potassium channel 2 (SK2) in IO ion channel studies (Fig. 7), Rat anti-SK2 primary Ab (1:200, NeuroMab clone K78/29, Cat# 75-403, RRID:AB_2877597) and Goat anti-Rat IgG (H + L) Alexa Fluor 488-conjugated secondary Ab (1:200, Invitrogen, Cat# A11006, RRID:AB_2534074) were used. To stain for Calbindin (Calb) for IO ion channel studies (Fig. 7), Mouse anti-Calb primary Ab (1:1000; Sigma Aldrich, Cat# C9848, RRID:AB_476894) and Goat anti-Mouse IgG (H + L) Alexa Fluor 594-conjugated secondary Ab (1:200; Invitrogen, Cat# A11005, RRID:AB_2534073) were used. VGLUT1 Polyclonal antibody: Invitrogen, Ref #PA5-142376, at a concentration of 1:500, VGLUT2 Polyclonal antibody: Invitrogen, Ref #42-7800, at a concentration of 1:500 were used for to label excitatory synaptic terminals. Western blot analysis for GAD65/67 was performed with a polyclonal antibody (1:1000, Invitrogen, ref#: PA1-84572) and GAPDH (1:4000, Protein Tech, cat#: 60004-I-Ig) was used as a loading control.

#### Fluorescence intensity measurements

To measure the intensity of GAD, VGLUT1 and VGLUT2 staining (Figs 3 and 4), images acquired at 10× magnification were used. Fluorescence intensity analysis was performed using ImageJ. A rectangular box was placed in the IO, specifically in the area of the principal olivary nucleus (IOPr). Mean pixel intensity was measured for each rectangle, which was then used as the raw fluorescence intensity value for each section. Box size was identical in all cases and placed in similar areas of the IOPr between sections. Two sections were imaged per animal and the mean of their fluorescence values were used as the fluorescence intensity for that animal. All tissue processing and imaging was performed at the same session and microscope settings were identical for all acquired images. During imaging and analysis, the experimenter was blind to genotype.

#### Confocal microscopy

Imaging was performed on a Nikon C2+ confocal microscope. Single-plane images were acquired at 63× magnification using an oil-immersion lens, with microscope settings kept constant between all samples under each set of antibodies. Samples were prepared and imaged with the experimenter blind to genotype.

### Inferior olive stereology

#### Cell counts

Estimates for the total number of neurons (NeuN^+^ cells) in the IO, total number of healthy neurons (NeuN^+^,Calb^+^ cells) in the IO, and total IO volume were quantified with an unbiased stereological approach using the optical fractionator probe in Stereoinvestigator (MBF Bioscience, Williston, VT). 30 μm serial coronal sections through the medulla were mounted on slides and co-stained for NeuN and Calbindin as described above. Sections were observed under epifluorescence on a Zeiss Axioimager M2 microscope, where regions of interest were first outlined using a 10× objective lens. 10 sections per brain were analyzed, with a section evaluation interval of 4. Cells within the outlined region were counted using a 63× oil immersion objective, with a ~20 μm counting depth and 1 μm guard zones. A 200 μm × 200 μm counting frame and 50 μm × 50 μm sampling grid were used for all counts, determined effective in pilot studies such that the Gunderson coefficient of error was less than 0.1 for all markers. The top of each stained cell body was the point of reference. The pyramids, ventral gigantocellular reticular nucleus, lateral paragigantocellular nucleus, medial lemniscus, and magnocellular reticular nucleus were used as anatomical boundaries for the IO.

#### Cell size measurements

IOPr neuron cell size was quantified by measuring the average soma area (μm^2^) for 30–50 cells per animal. Measurements were made concurrent with stereological analysis using the 63× oil immersion objective and the 4-ray nucleator probe in Stereoinvestigator (MBF Bioscience, Williston, VT). Cell size was measured while visualizing cells with NeuN. Analysis was constrained to the principal nucleus of the inferior olive (IOPr), identified by its distinct laminar structure. 200–400 neurons (from 5–8 brains) were measured for each genotype at each timepoint.

### Western blot analysis

Mice were deeply euthanized with isoflurane inhalation before decapitation. After removal of the cerebellum, a 3 mm segment of the brainstem medulla was isolated under magnification, flash-frozen in liquid nitrogen, and stored at −80°C for long-term use. Brainstem tissue was homogenized using Teflon tissue homogenizer tube in Igepal lysis buffer (50 mM Tris–HCl, 150 mM NaCl, 5 mM EDTA, 1 mM EGTA, pH 8.0, 1% Igepal CA-640 (Sigma-Aldrich, cat#: 4906845001) with protease (Sigma-Aldrich, cat#: 4693132001) and phosphatase (Roche, cat# 04906837) inhibitor added. Brain lysates were then centrifuged at 14 000 rpm for 20 min at 4°C in temperature. The supernatant was then aliquoted and stored at −80°C. Lysates were subsequently thawed on ice, and protein concentration was determined using a Bicinchoninic acid (BCA) protein assay kit (Pierce BCA Protein Assay Kit, cat#: 23227). Samples were prepared for loading with 30 μg of protein denatured in 2X Laemmli Sample Buffer (Bio-Rad, cat#: 1610737) with 5% of 2-Mercaptoethanol (Sigma, cat#: M6250) at 95°C for 5 min. Protein samples were loaded on 10% precast gel (Bio-Rad, cat#: 4561034). The gel was at 100 V and the resolved proteins were transferred on the polyvinylidene difluoride (PVDF) membrane, using Tris-Glycine buffer (Bio-Rad, cat#: 1610734) in 20% methanol for 1.5 to 2 h at a constant current of voltage of 100 V at 4°C. After transfer the membrane was blocked with 5% skimmed milk in TBS-T [Tris-buffered saline (TBS) and with tween 20 (T) (0.1% Tween 20)], for 1 h at room temperature (RT). The membrane was then incubated with primary antibodies, GAD65/67 (Invitrogen, ref#: PA1-84572) at 1:1000 dilution and GAPDH (Protein Tech, cat#: 60004-I-Ig) at 1:4000 dilution, overnight at 4°C in 5% BSA in TBS-T for immunoblot analysis. After primary antibody incubation, the membrane was washed 3 × 10 min each with TBS-T and then incubated with secondary antibodies in 5% skimmed milk for 1 h on rocker at RT. The secondary antibodies were removed, and the membrane was washed 3 × 10 min each with TBS-T. The blot was developed using the Western blotting substrate (Thermo Scientific, cat#: 32209) under the Chemi-Doc imaging system (Bio-Rad) and the densitometry was performed using the Image Lab software from Bio-Rad.

### Experimental design and statistical analysis

Experimenters were kept blind to genotype for all experiments to avoid biasing results. To prevent variability within experiments, every individual analysis was performed by the same experimenter (e.g. all GAD staining analysis was performed by the same person) and using the same conditions/settings (e.g. all staining for an experiment was done in a single session and analyzed with the same microscope settings) for that experiment’s full mouse cohort. Individual statistical tests are described in the figure legends for all data. As this was an exploratory study, the null hypotheses were not prespecified and calculations for statistical power were not performed prior to study initiation. It follows from the exploratory nature of the experiments that calculated *P* values cannot be interpreted as hypothesis testing but only as descriptive. Though formal power analysis was not performed, we estimated sample size based on our (and others’) previous work in SCA1 mouse models. Number of individual data points and total number of animals for all experiments are reported in figure legends. Statistical analysis was done using Microsoft Excel, Prism 6.0 (GraphPad Software, Boston, MA), and SigmaPlot (Systat Software, Palo Alto, CA), with statistical significance defined as *P* < 0.05. If statistical significance was achieved, we performed post-hoc analysis corresponding to the experiment, as specified, to account for multiple comparisons. All *t*-tests were two-tailed Student’s *t*-tests, with the level of significance (alpha) set at 0.05. Enrichment of ion channels was calculated using Fisher’s exact test, with initial enrichment calculated by comparing ion channel gene proportion amongst DEGs to ion channel gene proportion amongst all genes analyzed (270 potential mouse ion channel transcripts of 30 973 total transcripts assessed) in a 2 × 2 contingency table.

## Results

### Neurons of the principal olivary nucleus in SCA1 mice undergo hypertrophy

To assess the morphological changes of neurons in the principal olivary nucleus (IOPr), we filled individual IOPr neurons from coronal brainstem slices of 14-week-old SCA1 knock-in (SCA1-KI) mice. This mouse strain was used because it is the most genetically precise model of SCA1, generated by the insertion of 154 CAG repeats into the endogenous *Atxn1* locus. This causes slowly-progressing movement phenotypes that mirror human SCA1: SCA1-KI motor incoordination begins at 7–8 weeks [24], becomes robust by 14 weeks [39], and is severe by 30 weeks [40]. Importantly, these mice also model brainstem phenotypes (unlike other SCA1 mouse models), as they produce mutant ATXN1 in all cell types in which *Atxn1* is endogenously expressed.

**Figure 1:**
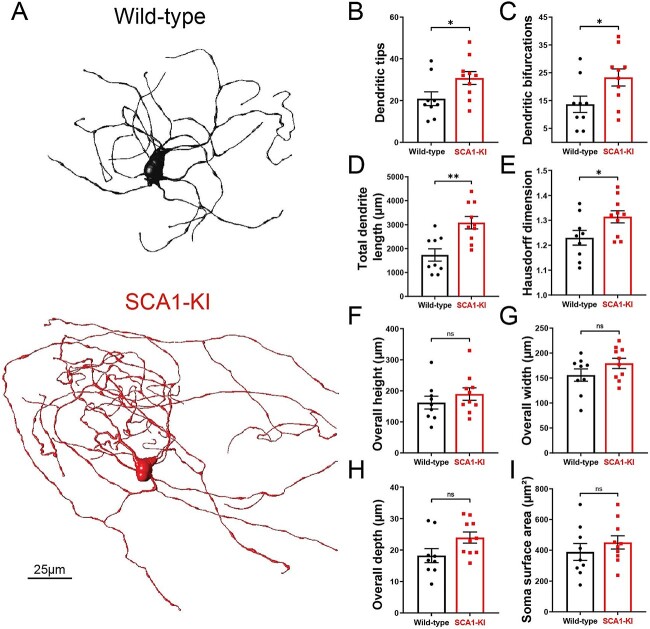
IOPr neuron dendritic arbors are hypertrophic in SCA1-KI mice. (A) Representative morphological reconstructions of dye-filled IO neurons in wild-type (top) and SCA1-KI (bottom) mice at approximately 14 weeks of age. (B–I) The dendritic arbors of SCA1-KI IO neurons are larger and more complex than wild-type controls. SCA1-KI IO neurons exhibit a greater number of dendritic tips (B) and dendritic bifurcations (C), as well as a marked increase in total dendritic length (D) and Hausdorff dimension (E) (a three-dimensional measure of object complexity). The overall height (F) and width (G) of IO neurons is largely unchanged between the two genotypes, though SCA1-KI IO neurons exhibit a significant increase in overall depth (H). No change in IO soma size (measured by surface area) was detected between the two genotypes (I). Data are expressed as mean ± SEM. Statistical significance derived by unpaired *t*-test with Welch’s correction, ^*^ = *P* < 0.05, ^*^^*^ = *P* < 0.01, ns = not significant.

Neurons of the IOPr are multipolar, having a “cloud” of dendrites surrounding the soma in three dimensions [2, 41]. At 14 weeks, we found that these IOPr dendritic arbors are significantly larger and more complex in SCA1-KI mice than in wild-type controls (Fig. 1A). SCA1-KI IOPr neurons have a greater number of dendritic tips and bifurcations (Fig. 1B and C), a larger total length of dendrites (Fig. 1D), and a significantly higher fractal dimensionality (a measure of the complexity of a three-dimensional object) (Fig. 1E). Though this indicates significant hypertrophy of SCA1-KI IOPr neuron dendrites, we did not observe a change in the dimensions of the volume element enclosing the dendritic arbor (Fig. 1F–H). Similarly, soma surface area was largely unchanged between genotypes (Fig. 1I).

**Figure 2:**
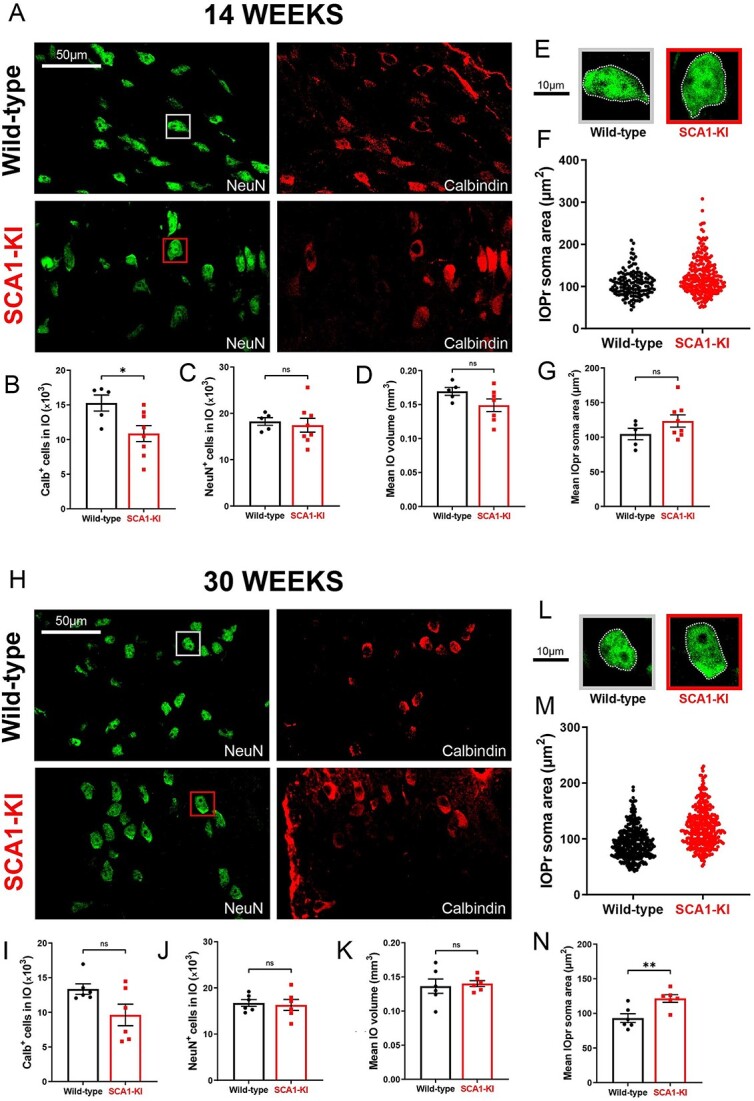
Inferior olive neurons in SCA1-KI mice undergo somatic hypertrophy. (A) Coronal histological sections showing IOPr neurons in wild-type (top) and SCA1-KI (bottom) mice at 14 weeks. Sections have been stained for NeuN and Calbindin. (B–D) Quantification of unbiased stereological analysis, providing an estimate of both total cell number and gross volume for the entire IO. At 14 weeks SCA1-KI IO neurons display a loss of calbindin immunoreactivity (B) without a change in NeuN^+^ cells (C) or IO volume (D). Inset showing representative IOPr cells of each genotype (E). (F) IOPr neuron soma size was estimated by measuring the area of the soma in ~200 cells per genotype. (G) No change in IOPr soma size in SCA1-KI mice is evident at the 14-week timepoint. (H) Coronal histological sections showing principal IO in wild-type (top) and SCA1-KI (bottom) mice at 30 weeks. Sections have been stained for NeuN and Calbindin. Quantification of unbiased stereological analysis reveals no statistically significant loss of Calb^+^, or NeuN^+^ cells (I and J) at 30 weeks. (K) Mean IO volume is preserved in SCA1-KI mice at 30 weeks. (L) Inset showing representative IOPr cells of each genotype. (M and N) IOPr neuron soma size was estimated by measuring the area of the soma in ~400 cells per genotype. At the 30-week timepoint, SCA1-KI IOPr neurons exhibit a shift in the entire distribution of neurons towards a larger size (M) with a highly significant increase in soma size (N). Data are expressed as mean ± SEM. Statistical significance derived by unpaired *t*-test with Welch’s correction, ^*^ = *P* < 0.05, ^*^^*^ = *P* < 0.01, ns = not significant.

To investigate SCA1-related phenotypes in the IO as a whole, we quantified IO cell number in 14-week and 30-week-old mice using unbiased stereology. This was performed using brainstem sections co-stained for neuronal nuclear protein (NeuN; a marker of all neurons) and calbindin (Calb). Calb is a well-conserved calcium-binding protein that plays a critical role in calcium buffering in Purkinje cells [42]. Historically, loss of Calb-immunoreactive (Calb^+^) Purkinje cells has been widely used as a surrogate for Purkinje cell degeneration in SCA mouse models, including models of SCA1 [24], SCA2 [43], SCA3 [44], and SCA17 [45]. Mouse IO neurons also exhibit high levels of Calb expression [25], and loss of Calb^+^ cells in the IO has similarly been used as an indicator of degeneration in the IO of both ataxic mice [46] and human SCA patients [23]. There was an overall reduction in Calb immunoreactivity in 14-week SCA1-KI IO (Fig. 2A). Using unbiased stereology in 14-week-old SCA1-KI mice, we identified a complete loss of Calb immunoreactivity in ~25% IO neurons (Fig. 2B) without a discernible decrease in total neurons, as evidenced by retention of the neuronal marker NeuN (Fig. 2C) or mean IO volume (Fig. 2D), suggesting the presence of early IO neurodegenerative changes in these mice. To assess cell size, we measured the soma area of 200–400 IOPr cells per genotype (Fig. 2E). At 14 weeks, we found no difference in the average soma area of IOPr neurons between genotypes (Fig. 2F and G), a result that is consistent with soma area measurements from filled cells at 14 weeks (Fig. 1I). At 30 weeks too, there was an overall reduction in calbindin reactivity (Fig. 2H). There was similarly lower Calb^+^ neurons in 30-week SCA1-KI mice compared to littermate controls as in 14-week SCA1-KI mice, although this did not reach statistical significance (*P* = 0.0658) (Fig. 2I). 30-week old animals also showed no difference in total IO cell number (Fig. 2J) or mean IO volume (Fig. 2K). At 30 weeks, IOPr soma areas were ~25% larger on average in SCA1-KI mice compared to wild-types (Fig. 2L–N), indicating significant somatic hypertrophy. These results, along with the morphological changes observed in single IOPr neurons at 14 weeks (Fig. 1), suggest an SCA1-associated hypertrophy in IOPr neurons that appears first in dendrites and later in the soma.

The concurrence of hypertrophy and degeneration is uncommon in neuronal disorders, but has been observed in the IO when brainstem injury (usually pontine hemorrhage [9]) causes substantive loss of inhibitory input to the IO [13], leading to hypertrophic olivary degeneration (HOD). The hypertrophy identified here appears strikingly similar to this well-described pathology, suggesting that SCA1-associated IO dysfunction may constitute a novel cause of olivary neuron hypertrophy (as seen in early stages of HOD).

### Hypertrophy in SCA1-KI IOPr neurons is not associated with a loss of inhibitory terminals

**Figure 3:**
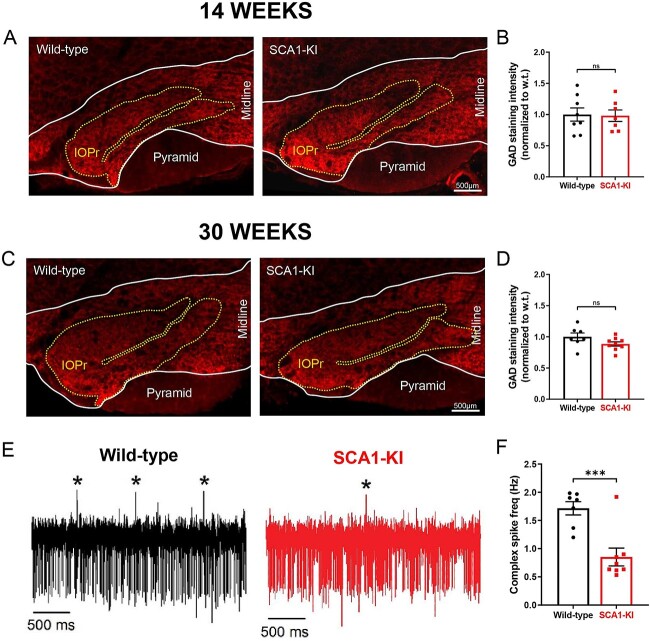
Inhibitory synaptic inputs remain intact in SCA1-KI inferior olive neurons. (A) Coronal histological sections showing the IOPr in wild-type (left) and SCA1-KI (right) mice at an early symptomatic timepoint of 14 weeks. Sections have been stained for glutamate decarboxylase (GAD), a marker of GABAergic terminals. (B) Quantification of staining reveals that GAD signal is retained in the SCA1-KI IO at 14 weeks. (C) Coronal histological sections showing GAD staining in the principal IO at a late symptomatic time point of 30 weeks. (D) Quantification of staining reveals that GAD signal is also retained in the SCA1-KI IO at 30 weeks. (E) Representative traces of *in-vivo* cerebellar spiking patterns in head-fixed, awake mice at 14 weeks. Complex spikes (generated by IO neurons) are indicated with asterisks (^*^). (F) Complex spike frequency is reduced in SCA1-KI cerebella, suggesting that IO neurons *in-vivo* are not disinhibited by loss of inhibitory synaptic input. Data are expressed as mean ± SEM. Statistical significance derived by unpaired *t*-test with Welch’s correction, ^*^^*^^*^ = *P* < 0.001, ns = not significant.

To rule out other potential causes of olivary hypertrophy, we analyzed inhibitory projections to the IOPr in SCA1-KI and wild-type mice at 14 weeks and 30 weeks. Coronal brainstem sections were stained for glutamate decarboxylase (GAD), a marker of GABAergic terminals (Fig. 3A and C). Prior work has indicated that a lesion to the cerebellar nuclei results in a near-complete loss of GAD-positive terminals in the IOPr, indicating that GAD is a good marker for the “intactness” of inhibitory innervation to the IOPr [47]. At both 14 weeks and 30 weeks, there was no reduction in the intensity of GAD staining between groups (Fig. 3B and D, Supplementary Fig. 1A), indicating that inhibitory input to the IOPr remains structurally intact during IO hypertrophy. Further, immunoblots from the brainstem medulla at 14-weeks failed to demonstrate a reduction in GAD65/67 (Supplementary Fig. 1B). To assess whether these inputs are functionally intact, we performed *in-vivo* recordings of cerebellar Purkinje cells (PCs) in awake, head-fixed SCA1-KI and wild-type mice at 14 weeks. The primary output of IO neurons is the complex spike (CS), a characteristic high-amplitude depolarization of the PC membrane that occurs when climbing fibers are activated [48, 49]. These CSs can be observed *in-vivo* as distinctly large depolarizing membrane deflections, as shown in our recordings (Fig. 3E). During these recordings, we observed significantly fewer CSs in SCA1-KI PCs compared to wild-type PCs (Fig. 3F, Supplementary Fig. 2). To determine if a reduction in excitatory terminals in the IO accounts for the reduction in complex spikes, we also examined changes in excitatory terminals in the IO using immunostaining for the vesicular glutamate transporters VGLUT1 and VGLUT2 in coronal brainstem sections in 14-week SCA1-KI mice. VGLUT1 and VGLUT2 staining were similar in SCA1-KI IO versus wild-type littermate controls (Supplementary Fig. 3). This suggests that SCA1-KI IOPr neurons are not experiencing a loss of inhibitory synaptic tone, as we would expect disinhibition of the IO to cause an increase in final IO output onto PCs (as previously described [50]).

Because SCA1-KI mice express mutant *Atxn1* via its endogenous promoter, ATXN1 function is altered broadly across the SCA1-KI mouse brain. Thus, it is possible that the observed IO hypertrophy is being influenced by other affected brain regions within the IO circuit. To determine whether mutant ATXN1-mediated dysfunction outside of the IO contributes to this degenerative hypertrophy, we assessed cell number, cell size, and the density of inhibitory inputs in SCA1 transgenic (SCA1-Tg) mice, which maintain normal ATXN1 expression in the IO. These mice overexpress the human *ATXN1* gene with an expanded CAG triplet repeat under the murine *Pcp2 (L7)* promoter [28], driving selective expression of polyglutamine-expanded ATXN1 (82 repeats) in cerebellar Purkinje cells. At 14 weeks, SCA1-Tg mice exhibit prominent cerebellar Purkinje cell atrophy without overt cell loss [51]. Similar to SCA1-KI mice [24], at 30 weeks, SCA1-Tg mice exhibit significant Purkinje cell loss [24, 52].

**Figure 4:**
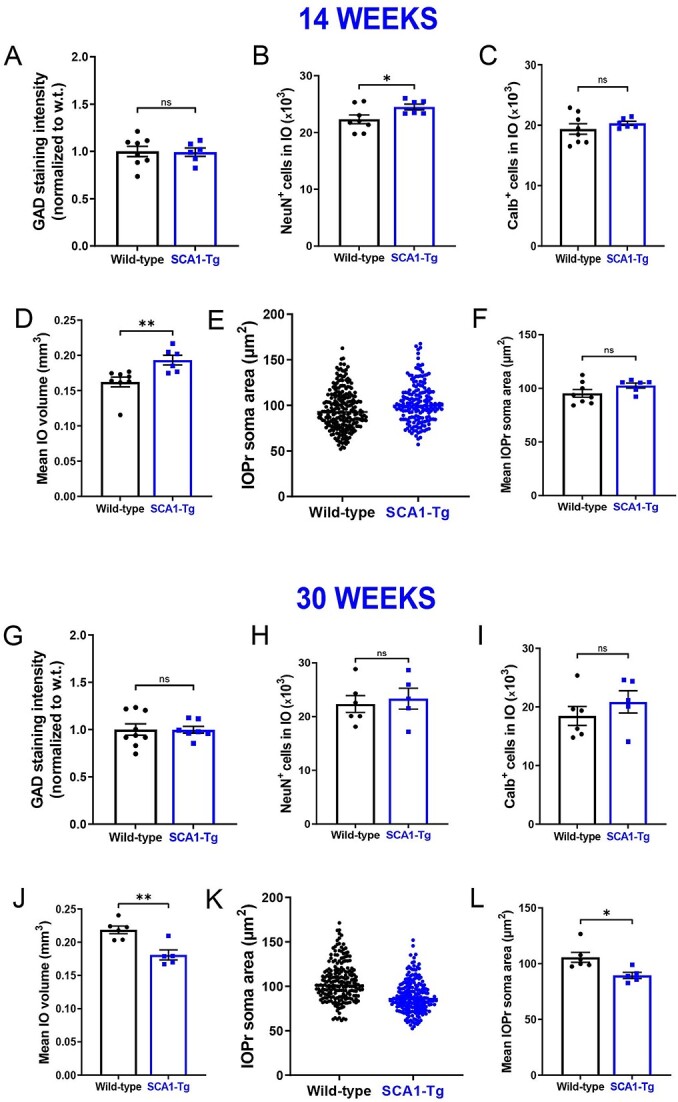
Olivary neuron hypertrophy in SCA1 mice is cell-autonomous. (A) Quantification of staining for GAD shows that signal is retained in the SCA1-Tg IO at 14 weeks, indicating no gross loss of inhibitory synaptic terminals in these mice. (B) Unbiased stereology of IO in SCA1-Tg mice at 14 weeks reveals a higher number of NeuN^+^ cells on the SCA1-KI IO compared to littermate controls. (C) There is no difference in number of Calb^+^ cells between SCA1-KI mice and littermate controls. (D) Mean IO volume was higher in SCA1-KI mice. (E and F) IOPr neuron soma size was estimated by measuring the area of the soma in ~200 cells per genotype. No change in IOPr soma size in SCA1-Tg mice is evident at this timepoint. (G) Quantification of staining for GAD shows that signal is also retained in the SCA1-Tg IO at 30 weeks. (H and I) Unbiased stereology of IO in SCA1-Tg mice at 30 weeks reveals no change in number of NeuN^+^ and Calb^+^ cells. (J) Quantification of unbiased stereological analysis, providing an estimate of both gross volume for the entire IO indicating that there is IO volume loss in the SCA1-Tg mice at 30 weeks. (K) IOPr neuron soma size was estimated by measuring the area of the soma in ~200 cells per genotype. A reduction in IOPr soma size in SCA1-Tg mice is evident at 30 weeks. (L). Data are expressed as mean ± SEM. Statistical significance derived by unpaired *t*-test with Welch’s correction, ^*^ = *P* < 0.05, ns = not significant.

IO GAD immunoreactivity was retained in SCA1-Tg mice at 14 weeks (Fig. 4A), suggesting that inhibitory terminals to the SCA1-Tg IO are structurally intact. Unbiased stereological quantification was also performed in the IO of SCA1-Tg mice. Although mean NeuN^+^ cell count (Fig. 4B) was higher, no signs of IO degeneration were evident at 14 weeks, indicated by the absence of a change in Calb^+^ cell number (Fig. 4C). Consistent with the higher number of NeuN^+^ neurons, the mean IO volume (Fig. 4D) was significantly greater in SCA1-Tg mice compared to wild-type littermate controls. IOPr soma size, however, was unchanged between genotypes (Fig. 4E and F). We further examined a later time point (30 weeks) in SCA1-Tg mice. At this time point, there was also no loss of GAD immunoreactivity (Fig. 4G) in the IO. Additionally, the number of NeuN^+^ and Calb^+^ cells in the IO was retained (Fig. 4H and I). Unexpectedly, mean IO volume (Fig. 4J) and IOPr soma size were both reduced (Fig. 4K and L) in SCA1-Tg mice compared to wild-type controls at 30 weeks. These results demonstrate that olivary atrophy without cell loss or Calb immunoreactivity changes can occur with alterations in the olivocerebellar circuit – even without direct mutant ATXN1 expression in the IO. Furthermore, it appears that olivary hypertrophy with loss of Calb-immunoreactive neurons only occurs when mutant ATXN1 expression is present in the IO (i.e., in SCA1-KI mice).

HOD has been historically understood as a non-cell-autonomous phenomenon, arising after inhibitory deafferentation of the IO [7, 16, 53, 54]. These results demonstrate that a similar cellular phenotype can occur in SCA1, associated with the absence of any overt changes in IO inhibitory tone or gross alterations in the number of excitatory or inhibitory synaptic terminals. Indeed, SCA1-Tg mice, which model a non-olivary disruption of the IO circuit, exhibit IO atrophy rather than hypertrophy. This reveals that the IO phenotype observed in SCA1-KI mice is likely cell-autonomous, suggesting a novel cause of olivary hypertrophy separate from any extrinsic source.

### SCA1-KI IOPr neurons are hyperexcitable

**Figure 5:**
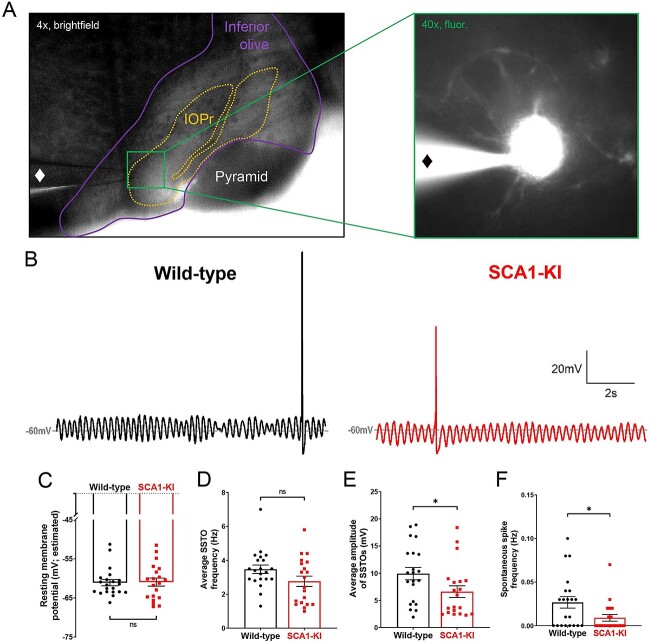
Spontaneous activity in SCA1-KI IOPr neurons is largely unchanged. (A) Images demonstrating whole-cell patch-clamp electrophysiology on IO neurons in coronal brainstem slices at 4× magnification (left) and 40× magnification (right). Internal solution included a small amount of fluorescent dye that filled cells while recording, allowing for the single-cell morphological analysis shown in Fig. 1. A ♦ symbol marks the recording electrode. (B) Representative traces of spontaneous activity in IOPr neurons from wild-type (left) and SCA1-KI (right) mice at 14 weeks. The presence of spontaneous subthreshold oscillations (SSTOs) was used as confirmation of cell type when recording. (C–F) Quantification of various electrophysiological properties show few differences between SCA1-KI and wild-type IOPr neurons at rest. There was no apparent change in resting membrane potential (C) or SSTO frequency (D) between genotypes, though SCA1-KI IOPr neurons did exhibit diminished SSTO amplitude (E) and spontaneous spike generation (F). Data are expressed as mean ± SEM. Statistical significance derived by unpaired *t*-test with Welch’s correction, ^*^ = *P* < 0.05, ns = not significant.

To explore potential intrinsic causes of olivary hypertrophy in SCA1, we performed patch-clamp electrophysiology on acute brainstem slices from 14-week-old SCA1-KI mice and wild-type littermate controls (Fig. 5A). All recordings were done in the IOPr, which was distinguished by its laminar structure and anatomical position lateral to the midline. The identity of targeted cells was confirmed by the presence of spontaneous subthreshold oscillations (SSTOs), a low-frequency fluctuation in membrane potential that has been well-established as a characteristic feature of IOPr neurons [55–57] (Fig. 5B). Using a whole-cell current clamp configuration, we observed the spontaneous activity of these neurons for ~100 s. Due to the persistence of SSTOs throughout these recordings, resting membrane potential could not be directly calculated; however, an estimate was made by taking the average potential across 10 s of oscillations (Fig. 5C). This estimate of resting membrane potential, as well as the average frequency of SSTOs (Fig. 5D), was not significantly different between SCA1-KI IOPr neurons and wild-type IOPr neurons. SCA1-KI IOPr neurons did, however, exhibit a decreased average SSTO amplitude (Fig. 5E) and spontaneous spike frequency (Fig. 5F).

**Figure 6:**
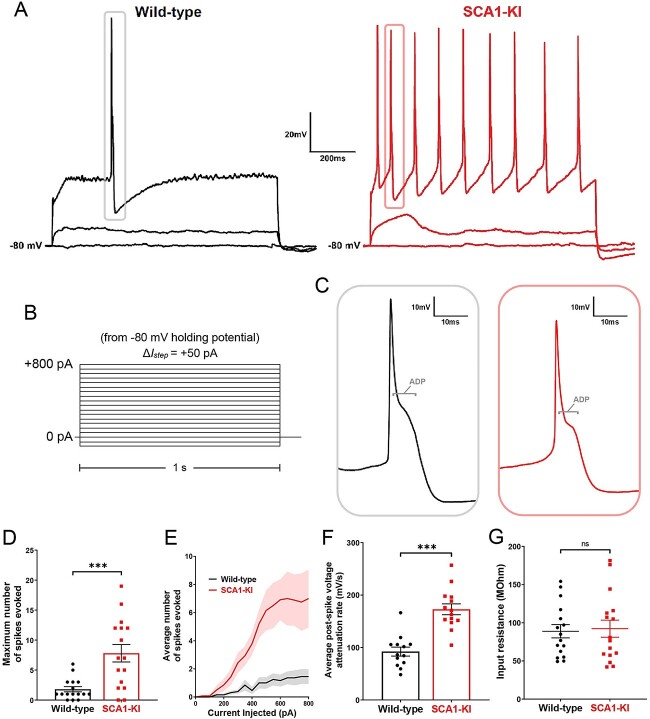
IOPr neurons in SCA1-KI mice are hyperexcitable. (A) Representative traces of evoked activity in IO neurons from wild-type (left) and SCA1-KI (right) mice at 14 weeks. Traces shown for each group are 0 pA injected (bottom trace), 200 pA injected (middle trace), and 550 pA injected (top trace). (B) From a holding potential of −80 mV, depolarizing currents were injected ranging from 50 pA to 800 pA in 50 pA increments and using 1 s steps. Recordings were performed in current clamp mode. (C) Inset of representative evoked spikes at a higher timescale resolution reveal a long afterdepolarization (ADP) “hump,” a characteristic feature of IOPr neurons. (D) Unlike IO neurons in wild-type mice, IO neurons in SCA1-KI mice are able to sustain a spike train. (E) Input–output curve of average spikes produced in IO neurons of each genotype. Number of spikes rose steadily with current injection in SCA1-KI IO neurons, while wild-type IO neurons rarely exhibited spiking in the range depicted (0-800 pA injected). (F) In SCA1-KI IO neurons, the afterhyperpolarization (AHP) of evoked spikes decayed more quickly, allowing these neurons to fire repetitively. (G) Input resistance of IO neurons is unchanged in SCA1-KI mice, demonstrating that this hyperexcitability phenotype is not a product of any change in voltage generated per injected current step. Data are expressed as mean ± SEM. Statistical significance derived by unpaired *t*-test with Welch’s correction, ^*^^*^^*^ = *P* < 0.001, ns = not significant.

After recording spontaneous activity in IOPr neurons, we assessed their evoked activity by injecting increasing levels of current. Preliminary tests revealed that holding cells at a slightly hyperpolarized potential (−80 mV) limited most active conductances. This allowed for the injection of current from a stable baseline, free of SSTOs, spontaneous spikes, and other potential confounding activity. From −80 mV, we injected 0–800 pA depolarizing current in repeated 1 s steps, increasing the amount injected by +50 pA with each successive sweep (Fig. 6B). The initial spike from −80 mV was a spike with a narrow half-width (so called “low-threshold” spike) that is less frequently encountered in IO neurons at rest [29, 30]. Subsequent spikes displayed the characteristically wide spikes previously described in IOPr neurons, with their distinctive large afterdepolarization (ADP) visible as a protruding “hump” in the repolarizing phase of the spike [29] (Fig. 6C). The analyses described below focused on these more typical IOPr events, excluding any initial low-threshold spikes.

Current injections in the IOPr revealed a novel hyperexcitability phenotype in the SCA1 IO (Fig. 6A). Unlike wild-type IOPr neurons, SCA1-KI IOPr neurons are able to fire repetitively (Fig. 6D) in spike trains that increase in frequency with greater injections of current (Fig. 6E). Input resistance was unchanged between the two genotypes (Fig. 6G), indicating that differences in excitability could not be explained by changes in passive membrane properties. The specific membrane resistance (calculated by input resistance/cell capacitance) was also similar between wild-type and SCA1-KI neurons (2.3 ± 0.4 MΩ/pF in wild-type, and 2.7 ± 0.5 MΩ/pF in SCA1-KI, student’s t-test, *P* > 0.05). Further analysis of these spikes suggests that the observed SCA1-KI IOPr hyperexcitability may be related to a disruption of post-spike conductances. In SCA1-KI mice, the rate at which IOPr membrane potential depolarizes back to baseline from its minimum is nearly 2-fold the rate observed in wild-type mice (Fig. 6F). This suggests that the membrane potential of SCA1-KI IOPr neurons can more quickly recover from a previous spike and reach threshold again, which may explain how these cells, unlike wild-type IOPr neurons, are able to generate spike trains.

### Ion channel transcripts are dysregulated in the SCA1-KI medulla

**Figure 7:**
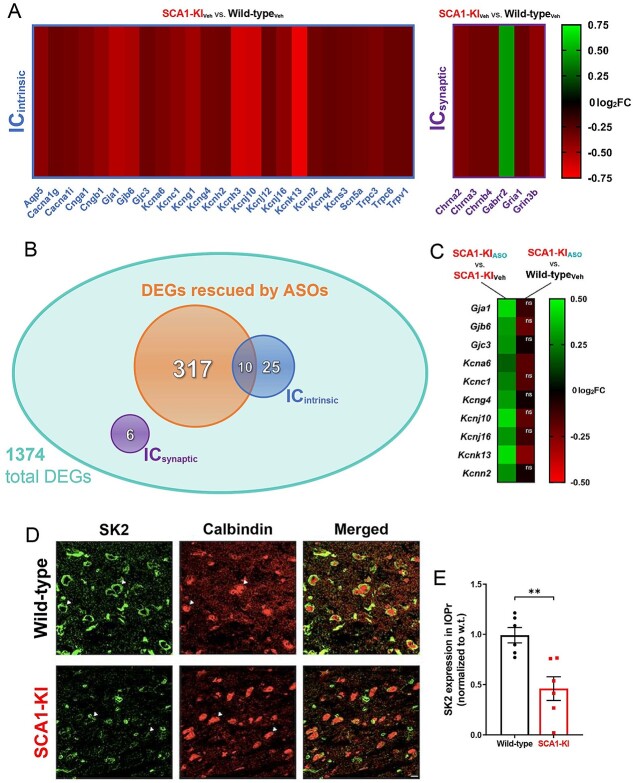
Ion channel expression is disrupted in the SCA1-KI medulla. (A) Transcriptomic analysis of whole medulla from SCA1-KI and wild-type mice at 28 weeks reveal that 31 ion channel genes are differentially expressed. Of these, 25 influence excitability intrinsically (IC_intrinsic_), while 6 influence excitability synaptically (IC_synaptic_). (B) Downregulation of *Atxn-1* expression by antisense oligonucleotides (ASOs) ameliorates brainstem phenotypes in SCA1-KI mice. Of the 1374 differentially expressed genes (DEGs) in the SCA1-KI medulla, 317 DEGs were significantly rescued by ASO treatment (and, therefore, are more likely to be responsible for the observed phenotypic rescue). Within this cohort, IC_intrinsic_ genes were significantly enriched. (C) Heatmap showing differential expression of the 10 IC_intrinsic_ DEGs between treatment groups. These ion channel genes exhibited a significant increase in expression after ASO treatment (left), with the majority of them rising to wild-type levels (right). (D) In order to examine how decreases in medullary ion channel transcripts may result in a loss of channels in the IOPr, immunostaining for the small-conductance potassium channel SK2 was performed. Coronal histological sections of the IOPr in wild-type (top) and SCA1-KI (bottom) mice at 14 weeks are shown. Sections have been stained for SK2 and calbindin. (E) Quantification of immunostaining reveals a significant loss of SK2 channels on the membrane of SCA1-KI IOPr neurons. Data are expressed as mean ± SEM. Statistical significance derived by Fischer’s exact test (enrichment analyses) or unpaired *t*-test with Welch’s correction (all other comparisons), ^*^^*^^*^ = *P* < 0.001, ns = not significant.

A prior study has examined alterations in transcripts in the medullary brainstem in SCA1-KI mice [58]. To assess potential causes of hyperexcitability in the SCA1-KI IO, we analyzed this previously-obtained medullary transcriptome data from SCA1-KI and wild-type controls. This study was also designed the test the efficacy of *Atxn1*-targeting antisense oligonucleotides (ASOs) (small molecules designed to block *Atxn1* expression) in improving behavioral outcomes. In this prior study, after being administered ASOs at 5 weeks of age, SCA1-KI mice demonstrated significant rescue in both motor behavior and brainstem phenotypes [58]. As part of this study, 28-week medullary tissue was harvested from both treated and non-treated mice, as well as untreated wild-type littermate controls, and analyzed by RNA-seq. Comparing untreated wild-type and untreated SCA1-KI medullary transcripts revealed significant dysregulation in 1374 genes [58]. We identified 31 ion channel transcripts among these baseline differentially-expressed genes (DEGs), representing a significant 2.79-fold enrichment compared to ion channel representation in the mouse genome (Fisher’s exact test, *P* = 1.41E-6), suggesting that disrupted ion channel expression could play a key role in the development of SCA1-KI brainstem phenotypes. Of these 31 candidate genes, 6 encode ion channels that influence excitability synaptically (IC_synaptic_), while the remaining 25 encode ion channels that influence excitability intrinsically (IC_intrinsic_) [59]. For all but one of these channels, medullary transcripts were downregulated in SCA1-KI mice compared to wild-type controls (Fig. 7A), a finding that comports with previous studies that report a primarily downregulated DEG population in SCA1 mouse models [60, 61].

To further narrow down this list of potential candidate genes, we analyzed data amongst the ASO treatment groups. Of the original 1374 medullary DEGs in SCA1-KI mice, 317 showed significant rescue after ASO treatment. Included among these 317 ASO-rescued DEGs were 10 ion channel genes, all of which encoded IC_intrinsic_ channels (Fig. 7B). This constituted a significant 5.26-fold enrichment of IC_intrinsic_ genes compared to IC_intrinsic_ representation in the mouse genome (Fisher’s exact test, *P* = 3.43E-5), suggesting that the loss of ion channels that regulate intrinsic excitability may be an important contributing factor to brainstem dysfunction in SCA1-KI mice. Of the IC_intrinsic_ candidate genes that underwent significant rescue by ASO treatment, the majority (8 of the 10) achieved “complete” rescue; that is, their expression level after ASO treatment was not significantly different from baseline levels in untreated wild-type mice (Fig. 7C). The full medullary transcriptome dataset analyzed here is available in Supplementary Table 1, which reports raw reads for all 30,973 transcripts assessed in each group, as well as the results of the statistical comparisons between groups that were used to define DEGs.

To determine whether this decrease in medullary IC_intrinsic_ transcripts reflects a loss of protein levels in the IO, we immunostained coronal brainstem slices from 30-week-old SCA1-KI and wild-type mice for the channel encoded by *Kcnn2*: small conductance calcium-activated potassium channel 2 (SK2) (Fig. 7D). Quantification of SK2 staining intensity on IOPr neurons revealed a ~50% reduction in protein levels (Fig. 7E). Taken together, these results suggest that downregulation of IC_intrinsic_ genes in the medulla may play a critical role in SCA1 brainstem pathogenesis.

### Spikes from SCA1-KI IOPr neurons exhibit a diminished AHP

**Figure 8:**
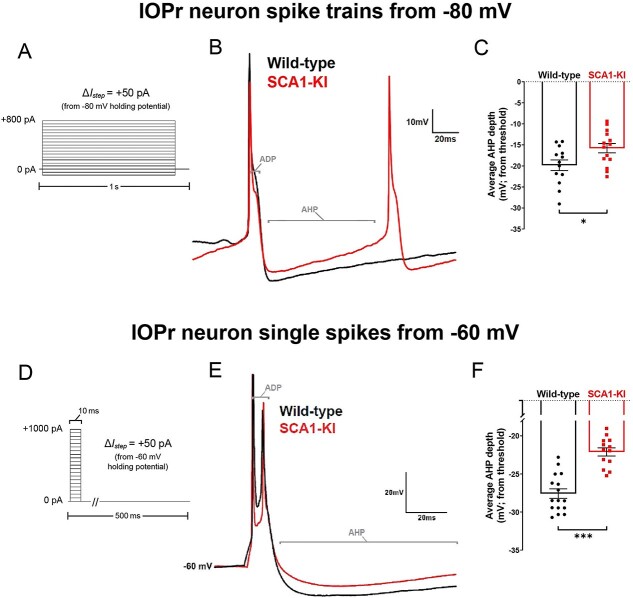
Spike afterhyperpolarization is diminished in SCA1-KI IOPr neurons. (A) In order to assess the functional relevance of ion channel loss in SCA1-KI IOPr neurons, spike trains were generated by injecting increasing levels of current from −80 mV (a holding potential at which active conductances are minimal). (B) Representative traces of spikes generated by injecting +450 pA current in IOPr neurons of wild-type and SCA1-KI mice at 14 weeks. (C) Quantification of AHP depth (measured from each spike’s threshold), demonstrating that SCA1-KI IOPr spikes during spike trains exhibit a shallower AHP compared to wild-type. (D) In order to determine if this AHP loss can also occur at rest, single spikes were generated by injecting increasing levels of current from −60 mV (a holding potential close to resting V_m_). Because the amount of current needed to generate a single spike varied between cells, the first spike that exhibited an ADP was used for comparisons. (E) Representative traces of evoked activity in IOPr neurons in wild-type and SCA1-KI mice at 14 weeks are overlaid. (F) Quantification of AHP depth (measured from baseline, −60 mV), demonstrating that SCA1-KI IO neuron spikes from rest also exhibit a shallower AHP compared to wild-type. Data are expressed as mean ± SEM. Statistical significance derived by unpaired *t*-test with Welch’s correction, ^*^ = *P* < 0.05, ^*^^*^^*^ = *P* < 0.001.

In order to assess the functional consequence of IC_intrinsic_ channel loss in the medulla, we recorded from IOPr neurons using patch-clamp electrophysiology in acute brainstem slices. Using the same current clamp protocol described above (Fig. 8A), increasing levels of current were injected from a holding potential of −80 mV in 1 s steps to generate spikes. AHP depth from each spike generated from 0 to 800 pA current injected was normalized to that spike’s threshold and compiled (note: two hyperpolarizing currents, −100 pA and −50 pA, were injected at the beginning of this protocol to allow for input resistance calculations before interference by spike generation). This revealed a significant loss of average AHP depth in SCA1-KI IOPr neurons (Fig. 8B and C). To determine whether this phenomenon is also capable of occurring in IOPr neurons at the resting membrane potential, we conducted similar spike analysis experiments, this time generating single spikes from −60 mV (a holding potential close to the estimated resting membrane potential of these cells (Fig. 5C)). In the whole-cell current clamp configuration, we injected 0–1000 pA depolarizing current in repeated 10 ms steps, increasing the amount injected by +50 pA with each successive sweep (Fig. 8D). In this setting, without the presence of spike trains, average AHP depth was again diminished in SCA1-KI IOPr neurons compared to wild-type IOPr neurons (Fig. 8E, F). Previous studies have demonstrated that potassium channels are the primary determinants of AHP size and shape in IOPr neurons. The observed AHP deficit in SCA1-KI IOPr neurons, as well as the enrichment of IC_intrinsic_ genes among ASO-rescued DEGs in the SCA1-KI medulla (7 of which encode potassium channels (Fig. 7C)), connects ion channel loss in the SCA1 brainstem to a potential functional consequence in IOPr hyperexcitability.

## Discussion

Though the IO has long been identified as a characteristic area of SCA1 pathology [8], it is not known what mechanisms drive disease in this cell population. Here, we describe hypertrophy in SCA1 IOPr neurons, a previously-unknown IO phenotype of the SCA1-KI mouse model. Additionally, we observe a loss of calbindin (Calb) immunoreactvity in SCA1 IO neurons. This may be suggestive of a degenerative process, as SCA1-KI mice also exhibit a loss of Calb immunoreactivity in cerebellar Purkinje cells prior to the loss of these neurons [24]. The hypertrophy observed in IOPr neurons of SCA1-KI mice appears similar to early stages of hypertrophic olivary degeneration (HOD), a pathology caused by the loss of inhibitory afferents to the IO [12, 13] that eventually leads to IO degeneration. Since SCA1-KI mice typically die by 35 weeks, we were unable to determine whether IO neuronal loss does eventually occur in SCA1-KI mice [24]. Dendritic hypertrophy in human HOD has been described (citation to thesis defense [62] in [17]) and, in a model of HOD after hemicerebellectomy in cats, olivary neurons display a greatly expanded dendritic tree, as well as somatic hypertrophy [12]. Taken together, these observations suggest that both dendritic and somatic hypertrophy occur during HOD.

Loss of inhibitory input from the cerebellar nucleus to the IO characteristically results in the loss of GAD-positive terminals to the IOPr [47]. In this study, we demonstrate that GAD-positive terminals representing inhibitory innervation to the IO in SCA1-KI mice are grossly intact, structurally. However, to definitively confirm that functional inhibitory deafferentation is absent in SCA1-KI mice, direct recordings of olivary inhibitory postsynaptic currents in brainstem slices are needed. Nevertheless, this work constitutes the first description of a robust HOD-like phenotype in the absence of prominent structural markers of inhibitory deafferentation in a spinocerebellar ataxia, and is consistent with previously-reported HOD in several individuals with SCA2 (diagnosed via brain MRI) [63]. Together, these results suggest that olivary hypertrophy is not solely the result of synaptic disinhibition and that there may be some downstream consequence of synaptic disinhibition, rather than synaptic disinhibition itself, that drives olivary hypertrophy.

Our work indicates that increased intrinsic excitability accompanies the olivary hypertrophy in SCA1-KI mice. One potential explanation for this connection between excitability and cell size is the possibility that, in the SCA1 IO, hypertrophy is acting in opposition to hyperexcitability as a compensatory mechanism. A substantial lengthening of dendrites, as exhibited in SCA1-KI mice at 14 weeks (Fig. 1), is likely to enhance dendritic shunting [64] of synaptic currents in the absence of a corresponding change in the number of synaptic contacts. If the multiple excitatory signals that the IO receives were sufficiently diluted by shunting through this extra length of dendrite, membrane excitability would, effectively, be decreased. Our finding of a lack of increase in excitatory VGLUT1 and VGLUT2 terminals in the olive is consistent with the idea that the increase in dendrite length is not associated with a corresponding increase in excitatory terminals, and that the observed reduction in complex spike frequency is a consequence of a passive increase in dendrite length. A caveat to this interpretation for the reduction in complex spikes is that changes in Purkinje cell intrinsic excitability alone can have consequences for the olivocerebellar circuit, resulting in a reduction in complex spike frequency [65]. For example, in a Purkinje-cell-specific BK channel knockout mouse, there is a reduction in complex spike frequency. The increase in excitability in response to depolarizing current injection that we observed in SCA1-KI IOPr neurons is likely, however, to not be the result of changes in the passive properties of the cell, as cat models of olivary hypertrophy after contralateral cerebellectomy exhibit a lower propensity to elicit high threshold calcium spikes with direct somatic IO depolarization [12]. This reduction in intrinsic excitability in inhibitory denervation-induced HOD is unlike the observed increase in high threshold spikes that we could elicit with somatic current injection in SCA1-KI IOPr neurons. These results suggest a complex relationship between morphology and excitability in IO neurons in SCA1, requiring further work in genetically-modified mice where the intrinsic and extrinsic mechanisms affecting neuronal excitability can be conditionally probed.

Previous research has demonstrated that the mutation that causes SCA1 (an expansion in the polyglutamine-encoding CAG repeat region of the *ATXN1* gene) produces Purkinje cell pathology by disrupting the expression of a host of downstream genes [60, 66]. Due to an abnormal interaction between the polyglutamine-expanded ATXN1 protein and Capicua (CIC), a transcriptional repressor, the vast majority of these Purkinje cell genes exhibit decreased expression in SCA1 [67, 68]. Ion channel genes, including many that are crucial for maintaining Purkinje cell excitability, are significantly enriched among this group [52, 69]. Similarly, in this study, we found a significant enrichment of ion channel genes among the medullary DEGs identified in 28-week-old SCA1-KI mice, the majority of which also exhibit decreased expression. Interestingly, cerebellar transcripts from these same 28-week-old SCA1-KI mice reveal that DEGs in the SCA1 cerebellum and the SCA1 medulla are indeed similar, but not identical [58]. Between these two tissues, 7 ion channel DEGs are shared: *Cacna1g*, *Gria1*, *Kcna6*, *Kcnc1*, *Kcnk13*, *Kcnn2*, and *Trpc3*, all exhibiting decreased expression in both brain regions. Future examination of ion channel protein levels (e.g., using immunoblots) will be needed to confirm that there is indeed a reduction in the channels that exhibit reduced medullary transcripts. Based on these observations, it appears that the cerebellum and brainstem may share a common mechanism of SCA1 pathology in reduced ion channel expression. This connection might be explained by the shared presence of CIC, which is moderately expressed in both the mouse cerebellum and the mouse IO [70]. The discrepancies between 28 week SCA1-KI medullary and cerebellar DEGs, as suggested by a recent study [71], may be due to the involvement of transcription factors other than CIC that are also affected by polyglutamine-expanded ATXN1. Further studies will be required to advance our knowledge of SCA1-associated transcriptional dysregulation in the IO. Specifically, tools like spatial transcriptomics would be useful in determining the specific transcriptomic profile of the SCA1 IO, as the data analyzed here came from RNA extracted from the whole medulla.

Although the relationship between changes in physiology and cell size remains unclear, prior research has demonstrated that firing abnormalities in SCA1-KI Purkinje cells precede degeneration [72]. Previous work has shown that addressing this by increasing specific potassium conductances with pharmacological agents can restore SCA1-KI Purkinje cell firing *in-vitro*. Additionally, chronic treatment of SCA1-KI mice with these same drugs not only improves motor function but, importantly, also slows the atrophy and subsequent degeneration of Purkinje cells [39, 73]. By demonstrating that a reduction in SCA1-KI Purkinje cell hyperexcitability is sufficient to partially rescue cell size phenotypes, these results suggest that SCA1-associated disruptions of membrane excitability may be an important driver of morphological changes in Purkinje cells; that is, hyperexcitability may be driving atrophy in the SCA1 cerebellum. The current work suggests that similar changes in physiology may underlie morphological changes in the SCA1 brainstem; that is, hyperexcitability may be driving hypertrophy in the SCA1 IO. Identifying the specific ion channels that underlie the intrinsic hyperexcitability in SCA1 IO neurons will be important to test whether activating the function or restoring channel expression may improve IO morphology and motor impairment in SCA1 mice.

## Supplementary Material

Supplementary_figures_composite_ddae146

Morrison_et_al_2024_Supplementary_Table_1_ddae146

## References

[1] Lang EJ, Apps R, Bengtsson F. et al. The roles of the Olivocerebellar pathway in motor learning and motor control. Cerebellum 2017;16:230–252. 27193702 10.1007/s12311-016-0787-8 PMC5116294

[2] De Zeeuw CI, Simpson JI, Hoogenraad CC. et al. Microcircuitry and function of the inferior olive. Trends Neurosci 1998;21:391–400. 9735947 10.1016/s0166-2236(98)01310-1

[3] Ausim Azizi S . And the olive said to the cerebellum: organization and functional significance of the olivo-cerebellar system. Neuroscientist 2007;13:616–625. 17911222 10.1177/1073858407299286

[4] Ito M . Error detection and representation in the olivo-cerebellar system. Front Neural Circuits 2013;7:1. 23440175 10.3389/fncir.2013.00001 PMC3579189

[5] Llinas RR . The olivo-cerebellar system: a key to understanding the functional significance of intrinsic oscillatory brain properties. Front Neural Circuits 2013;7:96. 24478634 10.3389/fncir.2013.00096 PMC3904115

[6] Konigsmark BW, Weiner LP. The olivopontocerebellar atrophies: a review. Medicine (Baltimore) 1970;49:227–242. 4910986 10.1097/00005792-197005000-00003

[7] Duvoisin RC . An apology and an introduction to the olivopontocerebellar atrophies. Adv Neurol 1984;41:5–12. 6496230

[8] Seidel K, Siswanto S, Brunt ER. et al. Brain pathology of spinocerebellar ataxias. Acta Neuropathol 2012;124:1–21. 22684686 10.1007/s00401-012-1000-x

[9] Smets G, Lambert J, Tijssen M. et al. The dentato-rubro-olivary pathway revisited: new MR imaging observations regarding hypertrophic olivary degeneration. Clin Anat 2017;30:543–549. 28247932 10.1002/ca.22866

[10] Ogut E, Armagan K, Tufekci D. The Guillain-Mollaret triangle: a key player in motor coordination and control with implications for neurological disorders. Neurosurg Rev 2023;46:181. 37468768 10.1007/s10143-023-02086-1

[11] Jellinger K . Hypertrophy of the inferior olives. Report on 29 cases. Z Neurol 1973;205:153–174. 4127009 10.1007/BF00316018

[12] Ruigrok TJ, de Zeeuw CI, Voogd J. Hypertrophy of inferior olivary neurons: a degenerative, regenerative or plasticity phenomenon. Eur J Morphol 1990;28:224–239. 2245132

[13] Wang H, Wang Y, Wang R. et al. Hypertrophic olivary degeneration: a comprehensive review focusing on etiology. Brain Res 2019;1718:53–63. 31026459 10.1016/j.brainres.2019.04.024

[14] Goto N, Kaneko M. Olivary enlargement: chronological and morphometric analyses. Acta Neuropathol 1981;54:275–282. 7270084 10.1007/BF00697000

[15] Pandey P, Westbroek EM, Gooderham PA. et al. Cavernous malformation of brainstem, thalamus, and basal ganglia: a series of 176 patients. Neurosurgery 2013;72:573–589 discussion 588-579. 23262564 10.1227/NEU.0b013e318283c9c2

[16] de Zeeuw CI, Ruigrok TJ, Schalekamp MP. et al. Ultrastructural study of the cat hypertrophic inferior olive following anterograde tracing, immunocytochemistry, and intracellular labeling. Eur J Morphol 1990;28:240–255. 2245133

[17] Verhaart WJ, Voogd J. Hypertrophy of the inferior olives in the cat. J Neuropathol Exp Neurol 1962;21:92–104. 13925442 10.1097/00005072-196201000-00008

[18] Marani E, Boesten AJ. Histochemistry of experimental hypertrophy of the inferior olive of the cat. J Anat 1979;129:203–205. 511765

[19] Koeppen AH . The neuropathology of the adult cerebellum. Handb Clin Neurol 2018;154:129–149. 29903436 10.1016/B978-0-444-63956-1.00008-4 PMC6279249

[20] Durr A . Autosomal dominant cerebellar ataxias: polyglutamine expansions and beyond. Lancet Neurol 2010;9:885–894. 20723845 10.1016/S1474-4422(10)70183-6

[21] Rub U, Schols L, Paulson H. et al. Clinical features, neurogenetics and neuropathology of the polyglutamine spinocerebellar ataxias type 1, 2, 3, 6 and 7. Prog Neurobiol 2013;104:38–66. 23438480 10.1016/j.pneurobio.2013.01.001

[22] Ashizawa T, Figueroa KP, Perlman SL. et al. Clinical characteristics of patients with spinocerebellar ataxias 1, 2, 3 and 6 in the US; a prospective observational study. Orphanet J Rare Dis 2013;8:177. 24225362 10.1186/1750-1172-8-177 PMC3843578

[23] Koeppen AH, Ramirez RL, Bjork ST. et al. The reciprocal cerebellar circuitry in human hereditary ataxia. Cerebellum 2013;12:493–503. 23389921 10.1007/s12311-013-0456-0 PMC3700561

[24] Watase K, Weeber EJ, Xu B. et al. A long CAG repeat in the mouse Sca1 locus replicates SCA1 features and reveals the impact of protein solubility on selective neurodegeneration. Neuron 2002;34:905–919. 12086639 10.1016/s0896-6273(02)00733-x

[25] Yu Y, Fu Y, Watson C. The inferior olive of the C57BL/6J mouse: a chemoarchitectonic study. Anat Rec (Hoboken) 2014;297:289–300. 24443186 10.1002/ar.22866

[26] Vig PJ, Subramony SH, Burright EN. et al. Reduced immunoreactivity to calcium-binding proteins in Purkinje cells precedes onset of ataxia in spinocerebellar ataxia-1 transgenic mice. Neurology 1998;50:106–113. 9443466 10.1212/wnl.50.1.106

[27] Koeppen AH . The pathogenesis of spinocerebellar ataxia. Cerebellum 2005;4:62–73. 15895563 10.1080/14734220510007950

[28] Burright EN, Clark HB, Servadio A. et al. SCA1 transgenic mice: a model for neurodegeneration caused by an expanded CAG trinucleotide repeat. Cell 1995;82:937–948. 7553854 10.1016/0092-8674(95)90273-2

[29] Llinas R, Yarom Y. Properties and distribution of ionic conductances generating electroresponsiveness of mammalian inferior olivary neurones in vitro. J Physiol 1981;315:569–584. 7310722 10.1113/jphysiol.1981.sp013764 PMC1249399

[30] Llinas R, Yarom Y. Electrophysiology of mammalian inferior olivary neurones in vitro. Different types of voltage-dependent ionic conductances. J Physiol 1981;315:549–567. 6273544 10.1113/jphysiol.1981.sp013763 PMC1249398

[31] Lefler Y, Yarom Y, Uusisaari MY. Cerebellar inhibitory input to the inferior olive decreases electrical coupling and blocks subthreshold oscillations. Neuron 2014;81:1389–1400. 24656256 10.1016/j.neuron.2014.02.032

[32] Huang S, Uusisaari MY. Physiological temperature during brain slicing enhances the quality of acute slice preparations. Front Cell Neurosci 2013;7:48. 23630465 10.3389/fncel.2013.00048 PMC3632751

[33] Dell'Orco JM, Wasserman AH, Chopra R. et al. Neuronal atrophy early in degenerative ataxia is a compensatory mechanism to regulate membrane excitability. J Neurosci 2015;35:11292–11307. 26269637 10.1523/JNEUROSCI.1357-15.2015 PMC4532759

[34] Heiney SA, Kim J, Augustine GJ. et al. Precise control of movement kinematics by optogenetic inhibition of Purkinje cell activity. J Neurosci 2014;34:2321–2330. 24501371 10.1523/JNEUROSCI.4547-13.2014 PMC3913874

[35] Warnaar P, Couto J, Negrello M. et al. Duration of Purkinje cell complex spikes increases with their firing frequency. Front Cell Neurosci 2015;9:122. 25918500 10.3389/fncel.2015.00122 PMC4394703

[36] Peng H, Bria A, Zhou Z. et al. Extensible visualization and analysis for multidimensional images using Vaa3D. Nat Protoc 2014;9:193–208. 24385149 10.1038/nprot.2014.011

[37] Peng H, Ruan Z, Long F. et al. V3D enables real-time 3D visualization and quantitative analysis of large-scale biological image data sets. Nat Biotechnol 2010;28:348–353. 20231818 10.1038/nbt.1612 PMC2857929

[38] Peng H, Tang J, Xiao H. et al. Virtual finger boosts three-dimensional imaging and microsurgery as well as terabyte volume image visualization and analysis. Nat Commun 2014;5:4342. 25014658 10.1038/ncomms5342 PMC4104457

[39] Bushart DD, Huang H, Man LJ. et al. A Chlorzoxazone-baclofen combination improves cerebellar impairment in spinocerebellar ataxia type 1. Mov Disord 2021;36:622–631. 33151010 10.1002/mds.28355 PMC7987844

[40] Cvetanovic M, Patel JM, Marti HH. et al. Vascular endothelial growth factor ameliorates the ataxic phenotype in a mouse model of spinocerebellar ataxia type 1. Nat Med 2011;17:1445–1447. 22001907 10.1038/nm.2494 PMC3287040

[41] Vrieler N, Loyola S, Yarden-Rabinowitz Y. et al. Variability and directionality of inferior olive neuron dendrites revealed by detailed 3D characterization of an extensive morphological library. Brain Struct Funct 2019;224:1677–1695. 30929054 10.1007/s00429-019-01859-z PMC6509097

[42] Schwaller B, Meyer M, Schiffmann S. 'New' functions for 'old' proteins: the role of the calcium-binding proteins calbindin D-28k, calretinin and parvalbumin, in cerebellar physiology. Studies with knockout mice. Cerebellum 2002;1:241–258. 12879963 10.1080/147342202320883551

[43] Hansen ST, Meera P, Otis TS. et al. Changes in Purkinje cell firing and gene expression precede behavioral pathology in a mouse model of SCA2. Hum Mol Genet 2013;22:271–283. 23087021 10.1093/hmg/dds427 PMC3526159

[44] Switonski PM, Szlachcic WJ, Krzyzosiak WJ. et al. A new humanized ataxin-3 knock-in mouse model combines the genetic features, pathogenesis of neurons and glia and late disease onset of SCA3/MJD. Neurobiol Dis 2015;73:174–188. 25301414 10.1016/j.nbd.2014.09.020

[45] Cui Y, Yang S, Li XJ. et al. Genetically modified rodent models of SCA17. J Neurosci Res 2017;95:1540–1547. 27859490 10.1002/jnr.23984 PMC5508981

[46] Zanjani H, Herrup K, Mariani J. Cell number in the inferior olive of nervous and leaner mutant mice. J Neurogenet 2004;18:327–339. 15370195 10.1080/01677060390449482

[47] Fredette BJ, Mugnaini E. The GABAergic cerebello-olivary projection in the rat. Anat Embryol (Berl) 1991;184:225–243. 1793166 10.1007/BF01673258

[48] Davie JT, Clark BA, Hausser M. The origin of the complex spike in cerebellar Purkinje cells. J Neurosci 2008;28:7599–7609. 18650337 10.1523/JNEUROSCI.0559-08.2008 PMC2730632

[49] Streng ML, Popa LS, Ebner TJ. Complex spike wars: a new hope. Cerebellum 2018;17:735–746. 29982917 10.1007/s12311-018-0960-3 PMC6208864

[50] Lang EJ, Sugihara I, Llinas R. GABAergic modulation of complex spike activity by the cerebellar nucleoolivary pathway in rat. J Neurophysiol 1996;76:255–275. 8836223 10.1152/jn.1996.76.1.255

[51] Clark HB, Burright EN, Yunis WS. et al. Purkinje cell expression of a mutant allele of SCA1 in transgenic mice leads to disparate effects on motor behaviors, followed by a progressive cerebellar dysfunction and histological alterations. J Neurosci 1997;17:7385–7395. 9295384 10.1523/JNEUROSCI.17-19-07385.1997 PMC6573461

[52] Chopra R, Bushart DD, Cooper JP. et al. Altered Capicua expression drives regional Purkinje neuron vulnerability through ion channel gene dysregulation in spinocerebellar ataxia type 1. Hum Mol Genet 2020;29:3249–3265. 32964235 10.1093/hmg/ddaa212 PMC7689299

[53] Boesten AJ, Voogd J. Hypertrophy of neurons in the inferior olive after cerebellar ablations in the cat. Neurosci Lett 1985;61:49–54. 4080260 10.1016/0304-3940(85)90399-4

[54] Ferrer I, Genis D, Davalos A. et al. The Purkinje cell in olivopontocerebellar atrophy. A Golgi and immunocytochemical study. Neuropathol Appl Neurobiol 1994;20:38–46. 7516051 10.1111/j.1365-2990.1994.tb00955.x

[55] Choi S, Yu E, Kim D. et al. Subthreshold membrane potential oscillations in inferior olive neurons are dynamically regulated by P/Q- and T-type calcium channels: a study in mutant mice. J Physiol 2010;588:3031–3043. 20547676 10.1113/jphysiol.2009.184705 PMC2956943

[56] Llinas R, Yarom Y. Oscillatory properties of Guinea-pig inferior olivary neurones and their pharmacological modulation: an in vitro study. J Physiol 1986;376:163–182. 3795074 10.1113/jphysiol.1986.sp016147 PMC1182792

[57] Llinas RR . Inferior olive oscillation as the temporal basis for motricity and oscillatory reset as the basis for motor error correction. Neuroscience 2009;162:797–804. 19393291 10.1016/j.neuroscience.2009.04.045 PMC2861300

[58] Friedrich J, Kordasiewicz HB, O'Callaghan B. et al. Antisense oligonucleotide-mediated ataxin-1 reduction prolongs survival in SCA1 mice and reveals disease-associated transcriptome profiles. JCI Insight 2018;3:e123193. 10.1172/jci.insight.123193 PMC6238731 30385727

[59] Alexander SP, Mathie A, Peters JA. Guide to receptors and channels (GRAC), 5th edition. Br J Pharmacol 2011;164:S1–S324. 22040146 10.1111/j.1476-5381.2011.01649_1.x PMC3315626

[60] Lin X, Antalffy B, Kang D. et al. Polyglutamine expansion down-regulates specific neuronal genes before pathologic changes in SCA1. Nat Neurosci 2000;3:157–163. 10649571 10.1038/72101

[61] Niewiadomska-Cimicka A, Hache A, Trottier Y. Gene deregulation and underlying mechanisms in spinocerebellar ataxias with Polyglutamine expansion. Front Neurosci 2020;14:571. 32581696 10.3389/fnins.2020.00571 PMC7296114

[62] Perrelle-Aujard MDdl . Contribution a l'Etude anatomoclinique du nystagmus du voile et des myorythmies associees. D.P. Taib (thesis), Faculté de Médecine de Paris, Paris, FR, 1955;1–134.

[63] Yoshii F, Tomiyasu H, Watanabe R. et al. MRI signal abnormalities of the inferior Olivary nuclei in spinocerebellar ataxia type 2. Case Rep Neurol 2017;9:267–271. 29422848 10.1159/000481303 PMC5803720

[64] Blomfield S . Arithmetical operations performed by nerve cells. Brain Res 1974;69:115–124. 4817903 10.1016/0006-8993(74)90375-8

[65] Chen X, Kovalchuk Y, Adelsberger H. et al. Disruption of the olivo-cerebellar circuit by Purkinje neuron-specific ablation of BK channels. Proc Natl Acad Sci USA 2010;107:12323–12328. 20566869 10.1073/pnas.1001745107 PMC2901450

[66] Crespo-Barreto J, Fryer JD, Shaw CA. et al. Partial loss of ataxin-1 function contributes to transcriptional dysregulation in spinocerebellar ataxia type 1 pathogenesis. PLoS Genet 2010;6:e1001021. 20628574 10.1371/journal.pgen.1001021 PMC2900305

[67] Lam YC, Bowman AB, Jafar-Nejad P. et al. ATAXIN-1 interacts with the repressor Capicua in its native complex to cause SCA1 neuropathology. Cell 2006;127:1335–1347. 17190598 10.1016/j.cell.2006.11.038

[68] Rousseaux MWC, Tschumperlin T, Lu HC. et al. ATXN1-CIC complex is the primary driver of cerebellar pathology in spinocerebellar ataxia type 1 through a gain-of-function mechanism. Neuron 2018;97:1235–1243.e5. 29526553 10.1016/j.neuron.2018.02.013 PMC6422678

[69] Bushart DD, Chopra R, Singh V. et al. Targeting potassium channels to treat cerebellar ataxia. Ann Clin Transl Neurol 2018;5:297–314. 29560375 10.1002/acn3.527 PMC5846455

[70] Lein ES, Hawrylycz MJ, Ao N. et al. Genome-wide atlas of gene expression in the adult mouse brain. Nature 2007;445:168–176. 17151600 10.1038/nature05453

[71] Driessen TM, Lee PJ, Lim J. Molecular pathway analysis towards understanding tissue vulnerability in spinocerebellar ataxia type 1. elife 2018;7:e39981. 10.7554/eLife.39981 PMC6292693 30507379

[72] Hourez R, Servais L, Orduz D. et al. Aminopyridines correct early dysfunction and delay neurodegeneration in a mouse model of spinocerebellar ataxia type 1. J Neurosci 2011;31:11795–11807. 21849540 10.1523/JNEUROSCI.0905-11.2011 PMC6623197

[73] Chopra R, Bushart DD, Shakkottai VG. Dendritic potassium channel dysfunction may contribute to dendrite degeneration in spinocerebellar ataxia type 1. PLoS One 2018;13:e0198040. 29847609 10.1371/journal.pone.0198040 PMC5976172

