# Supplementary material for: Increased intrinsic membrane excitability is associated with olivary hypertrophy in spinocerebellar ataxia type 1
Source: Hum Mol Genet. 2024 Oct 30;33(24):2159–76. doi: 10.1093/hmg/ddae146 (PMC11630738; doi:10.1093/hmg/ddae146)
Supplement: Supplementary_figures_composite_ddae146 [file supplementary_figures_composite_ddae146.pdf]

Supplementary Figure 1

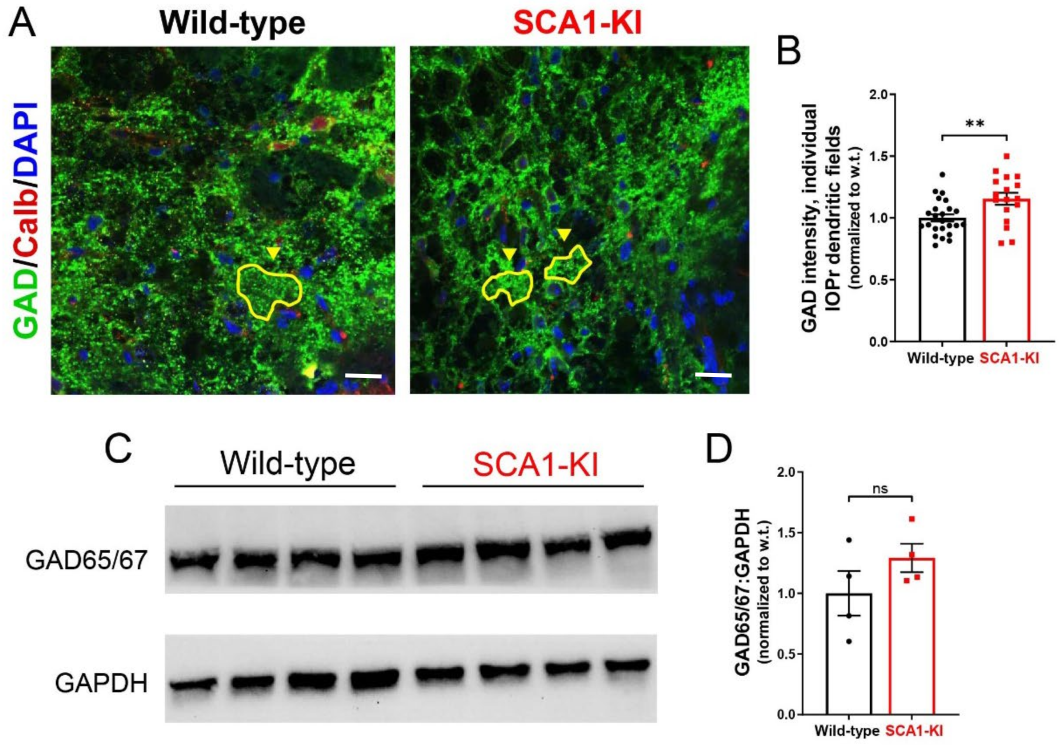

Supplementary Figure 1: Inhibitory terminals are intact in the inferior olivary nucleus in SCA1-KI mice. **(A)** Confocal images of IO neurons showing the dendritic field from individual neurons that was quantified for GAD immunoreactivity and quantified on the right (**B**). (**C**) Immunoblots for GAD from the medullary brainstem of wild-type and SCA1-KI mice showing no loss of GAD, summarized on the right (**D**). ** = $p < 0.01$, ns = not significant. Scale bars = 20 μm.

Supplementary Figure 2

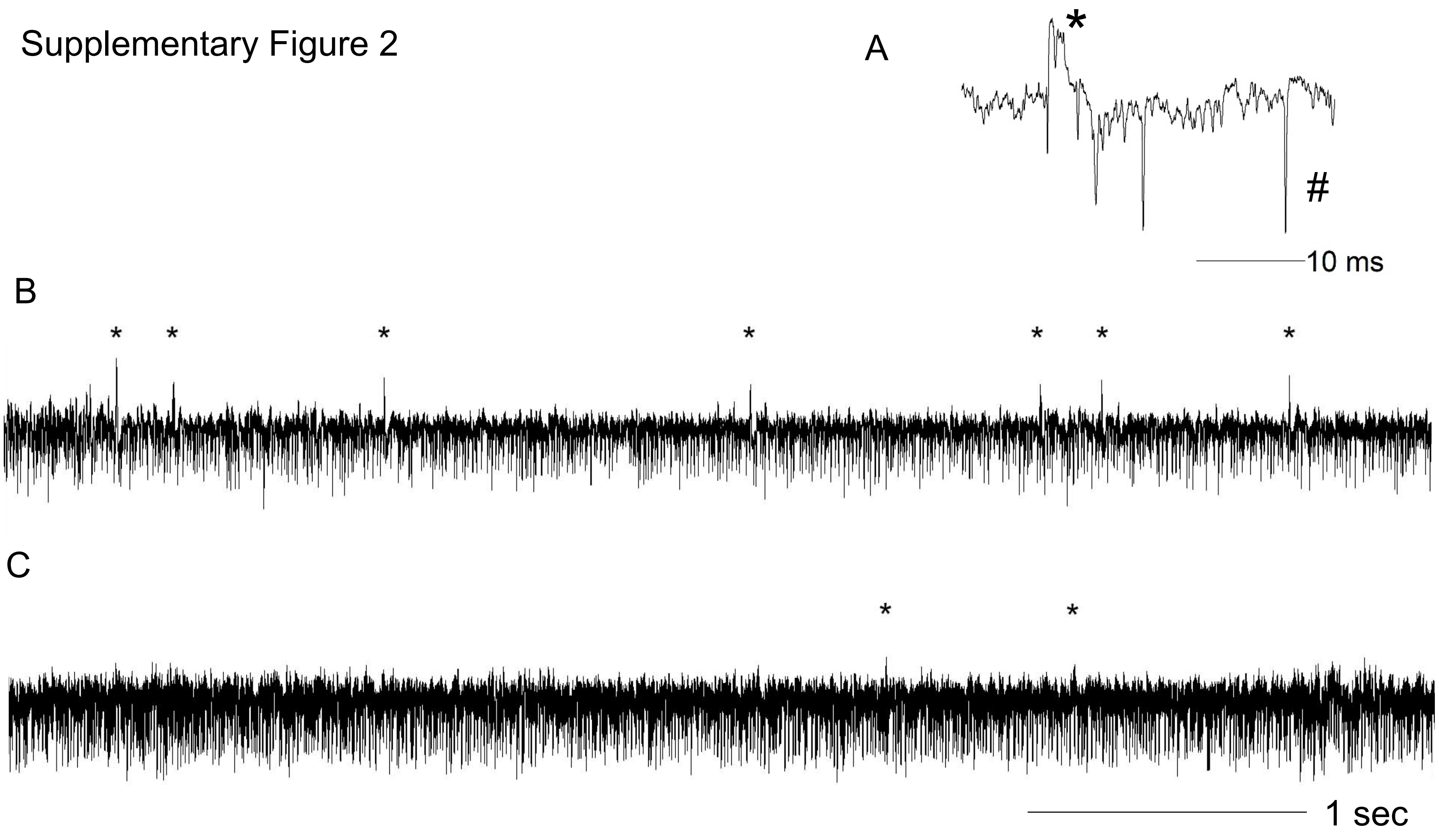

Supplementary Figure 2: Complex spike frequency is reduced in SCA1-KI mice. **(A)** *In vivo* recordings from awake head fixed wild-type mice at 14 weeks showing the characteristic upward deflection of the voltage from a complex spike (*). Simple spikes appear as a downward deflection of the voltage (#). (**B**) Recordings from wild-type mice at 14 weeks on a more expanded timescale showing the brief pause following each complex spike (*). (**C**) Complex spike (*) frequency is reduced in SCA1-KI mice. The pause in simple spikes is not as evident in SCA1-KI mice compared to wild-type littermate controls.

Supplementary Figure 3

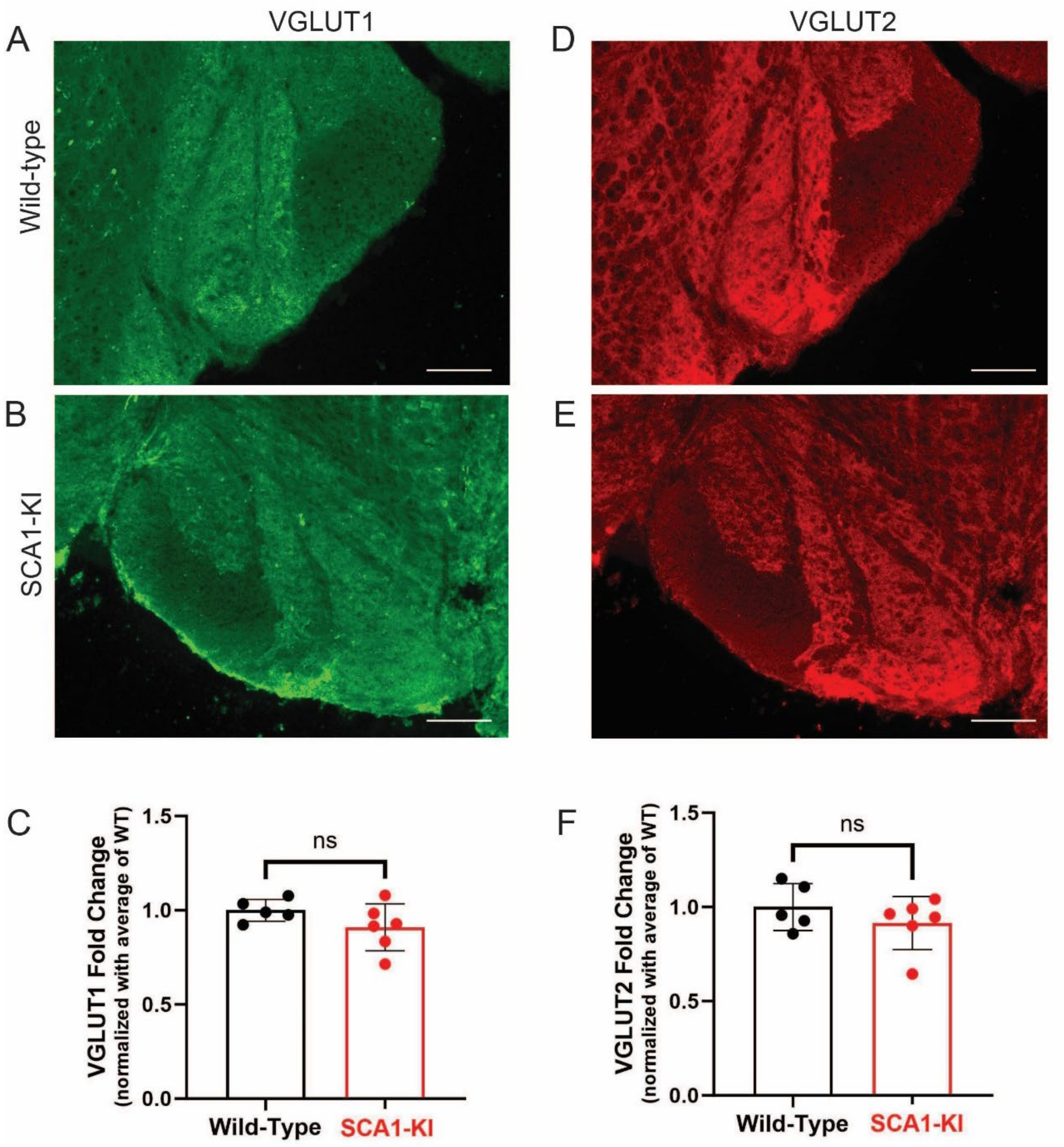

Supplementary Figure 3: Excitatory synaptic inputs are intact in SCA1-KI inferior olive neurons. **(A)** Coronal histological sections showing the IOPr in wild-type mice and SCA1-KI mice (**B**) at 14 weeks. Sections have been stained for vesicular glutamate transporter type 1 (green), a marker of excitatory terminals. (**C**) Quantification of staining reveals that VGLUT1 signal is retained in the SCA1-KI IO at 14 weeks. Unpaired t-test, ns = not significant. **(D)** Coronal histological sections showing the IOPr in wild-type mice and SCA1-KI mice (**E**) at 14 weeks. Sections have been stained for vesicular glutamate transporter type 2 (red), a marker of excitatory terminals. (**F**) Quantification of staining reveals that VGLUT2 signal is retained in the SCA1-KI IO at 14 weeks. Unpaired t-test, ns = not significant. Scale bars = 500 μM.
